# A new approach fits multivariate genomic prediction models efficiently

**DOI:** 10.1186/s12711-022-00730-w

**Published:** 2022-06-17

**Authors:** Alencar Xavier, David Habier

**Affiliations:** 1 Biostatistics, Corteva Agrisciences, 8305 NW 62nd Ave, Johnston, IA 50131 USA; 2 Department of Agronomy, Purdue University, 915 W State St, West Lafayette, IN 47907 USA

## Abstract

**Background:**

Fast, memory-efficient, and reliable algorithms for estimating genomic estimated breeding values (GEBV) for multiple traits and environments are needed to make timely decisions in breeding. Multivariate genomic prediction exploits genetic correlations between traits and environments to increase accuracy of GEBV compared to univariate methods. These genetic correlations are estimated simultaneously with GEBV, because they are specific to year, environment, and management. However, estimating genetic parameters is computationally demanding with restricted maximum likelihood (REML) and Bayesian samplers, and canonical transformations or orthogonalizations cannot be used for unbalanced experimental designs.

**Methods:**

We propose a multivariate randomized Gauss–Seidel algorithm for simultaneous estimation of model effects and genetic parameters. Two previously proposed methods for estimating genetic parameters were combined with a Gauss–Seidel (GS) solver, and were called *Tilde-Hat*-GS (THGS) and *Pseudo-Expectation*-GS (PEGS). Balanced and unbalanced experimental designs were simulated to compare runtime, bias and accuracy of GEBV, and bias and standard errors of estimates of heritabilities and genetic correlations of THGS, PEGS, and REML. Models with 10 to 400 response variables, 1279 to 42,034 genetic markers, and 5990 to 1.85 million observations were fitted.

**Results:**

Runtime of PEGS and THGS was a fraction of REML. Accuracies of GEBV were slightly lower than those from REML, but higher than those from the univariate approach, hence THGS and PEGS exploited genetic correlations. For 500 to 600 observations per response variable, biases of estimates of genetic parameters of THGS and PEGS were small, but standard errors of estimates of genetic correlations were higher than for REML. Bias and standard errors decreased as sample size increased. For balanced designs, GEBV and estimates of genetic correlations from THGS were unbiased when only an intercept and eigenvectors of genotype scores were fitted.

**Conclusions:**

THGS and PEGS are fast and memory-efficient algorithms for multivariate genomic prediction for balanced and unbalanced experimental designs. They are scalable for increasing numbers of environments and genetic markers. Accuracy of GEBV was comparable to REML. Estimates of genetic parameters had little bias, but their standard errors were larger than for REML. More studies are needed to evaluate the proposed methods for datasets that contain selection.

**Supplementary Information:**

The online version contains supplementary material available at 10.1186/s12711-022-00730-w.

## Background

Genomic prediction [1] uses genetic markers across the genome to predict complex diseases in humans and breeding values in animals and plants [2, 3]. Contrary to univariate analyses, multivariate genomic prediction [4] exploits genetic correlations among response variables to increase prediction accuracy for each variable [5]. In plant breeding, these response variables come from different quantitative traits that are measured in different field locations and years. Variance components and genetic correlations are estimated simultaneously with breeding values, because they vary across years, locations, and management. In animal breeding, in contrast, variance components are estimated infrequently within a breeding program and are used to solve mixed-model equations repeatedly over years.

Estimation of variances and covariances can be computationally demanding with standard multivariate approaches for trials with multiple quantitative traits and environments. In restricted maximum likelihood (REML) analyses, large and dense mixed-model equations need to be stored in memory and inverted repeatedly. In Bayesian analyses, model effects need to be sampled for thousands of Markov chain Monte Carlo (MCMC) iterations. This becomes time-consuming with an increasing number of response variables, because increasingly large matrices need to be inverted and factorized in each iteration. Canonical transformation [6] or diagonalization of genomic relationship matrices [7] can only be applied to balanced experimental designs when individuals are phenotyped in all environments and for all quantitative traits. However, unbalanced experimental designs are common. A solution would be to estimate genetic correlations for pairs of environments using bivariate models, but this also requires considerable computation resources. Moreover, the heritabilities of harvest yield are often low (0.1–0.2), so that the precision of estimated variance components for yield can be increased by analyzing yield together with higher heritable traits.

Fast and reliable algorithms are economically important in plant breeding enterprises to make timely decisions and advance the breeding pipeline. With any kind of delays during harvest season, e.g., due to weather, only a few hours may be available for selection decisions. If a breeder misses a deadline to request either new breeding crosses from nurseries or seed of selected individuals or seed of test-crosses, the generation interval increases, genetic gain per year decreases, and product launches are delayed.

To speed up computations and provide estimated breeding values on time, we propose to combine a randomized Gauss–Seidel [8, 9] solver for updating the effects of a multivariate model with an efficient approach for updating variances and covariances in each iteration of the algorithm. This approach calculates quadratic forms of random effects that resemble those used in REML but are equated to expectations that are easier to compute, as first proposed by [10, 11]. Similar approximations have been proposed over the years, as depicted in [12], who compared their *Tilde-Hat* approach to the methods of Schaeffer [13] and Henderson [14].

Statistical models that fit either a genomic relationship matrix or marker effects have been proposed for genomic prediction [2]. The latter is favored when the number of individuals exceeds the number of markers. In closed breeding programs, effective population sizes are such that a moderate number of markers, e.g. 10,000, is sufficient to estimate breeding values using training datasets with a larger number of individuals, e.g. 100,000.

The objective of this study was to present and evaluate a multivariate ridge regression approach that uses jointly a randomized Gauss–Seidel solver to estimate marker effects and the methods of either VanRaden [12] or Schaeffer [13] to estimate variances and covariances. Bias and accuracy of genomic estimated breeding values (GEBV) and runtime were studied by simulation of different scenarios, using a wheat dataset from CIMMYT’s Global Wheat Program and a soybean dataset from the SoyNAM project. The proposed methods were compared to standard software implementations of REML and univariate analyses to show that the approximations harness the benefits of multivariate models for prediction accuracy. Bayesian Gibbs sampling was added to compare runtime. To understand and interpret differences in bias and accuracy of GEBV between methods, biases and standard errors of estimates of heritabilities and genetic correlations were evaluated.

## Methods

### Statistical model

The multivariate ridge regression model can be written as

1 $${\mathbf {y}} = {\mathbf {X}}{\mathbf {b}} + {\mathbf {Z}}\varvec{\upbeta } + {\mathbf {e}},$$

where ${\mathbf {y}}$ is a vector of phenotypes from *K* environments, which can be partitioned into ${\mathbf {y}}^{\prime} = [{\mathbf {y}}^{\prime}_1 ~ {\mathbf {y}}^{\prime}_2 ~ \ldots {\mathbf {y}}^{\prime}_K]$, and each vector ${\mathbf {y}}^{\prime}_k$ has length $n_k$; ${\mathbf {X}} = \oplus _{k=1}^{K}{\mathbf {X}}_k$, $\oplus$ denotes the direct sum operator, ${\mathbf {X}}_k$ is an $n_k$ $\times$ $r_k$ matrix with full column rank of $r_k$ fixed effects; ${\mathbf {b}}^{\prime} = [{\mathbf {b}}^{\prime}_1 ~ {\mathbf {b}}^{\prime}_2 ~ \ldots {\mathbf {b}}^{\prime}_K]$ is a vector of fixed effects for all environments, and each vector ${\mathbf {b}}^{\prime}_k$ has length $r_k$; ${\mathbf {Z}} = \oplus _{k=1}^{K}{\mathbf {Z}}_k$, ${\mathbf {Z}}_k$ is an $n_k$ $\times$ *m* matrix that contains marker scores of $n_k$ individuals with phenotypes in environment *k* and *m* markers; $\varvec{\upbeta }^{\prime} = [\varvec{\upbeta }^{\prime}_1 ~ \varvec{\upbeta }^{\prime}_2 ~ \ldots \varvec{\upbeta }^{\prime}_K]$ is an ($m\cdot K$)-vector of random marker effects for all environments, and each vector $\varvec{\upbeta }^{\prime}_k$ has length *m*; ${\mathbf {e}}^{\prime} = [{\mathbf {e}}^{\prime}_1 ~ {\mathbf {e}}^{\prime}_2 ~ \ldots {\mathbf {e}}^{\prime}_K]$ is a vector of residuals, and each vector ${\mathbf {e}}^{\prime}_k$ has length $n_k$. Marker effects are assumed to be multivariate-normal distributed with mean zero and variance-covariance matrix $Var(\varvec{\upbeta }) = \varvec{\Sigma }_{\upbeta }\otimes {\mathbf {I}}_{m}$, where $\varvec{\Sigma }_{\upbeta }$ is a $K$ $\times$ $K$ matrix of genetic variances of marker effects, $\sigma ^2_{\upbeta _k}$, on the diagonal, and genetic covariances between marker effects from different environments, $\sigma _{\upbeta _{kk^{\prime}}}$, on the off-diagonal, $\otimes$ is the Kronecker product operator, and ${\mathbf {I}}_m$ is an identity matrix of dimension *m*. Residuals are assumed to be uncorrelated between environments, and normally distributed with mean zero and variance $Var({\mathbf {e}}) = \oplus _{k=1}^K{\mathbf {I}}_k\sigma ^2_{e_k}$.

### Solving fixed effects and marker effects

The mixed-model equations can be written as:

$$\begin{bmatrix} {\mathbf {X}}^{\prime}_1{\mathbf {X}}_1\sigma ^{-2}_{e_{1}} & \ldots & {\mathbf {0}} & {\mathbf {X}}^{\prime}_{1}{\mathbf {Z}}_{1}\sigma ^{-2}_{e_{1}} & \ldots & {\mathbf {0}}\\ \vdots & \ddots & \vdots & \vdots & \ddots & \vdots \\ {\mathbf {0}} & \ldots & {\mathbf {X}}^{\prime}_{K}{\mathbf {X}}_{K}\sigma ^{-2}_{e_{K}} & {\mathbf {0}} & \ldots & {\mathbf {X}}^{\prime}_{K}{\mathbf {Z}}_{K}\sigma ^{-2}_{e_{K}}\\ {\mathbf {Z}}^{\prime}_{1}{\mathbf {X}}^{\prime}_{1}\sigma ^{-2}_{e_{1}} & \ldots & {\mathbf {0}} & {\mathbf {Z}}^{\prime}_{1}{\mathbf {Z}}_{1}\sigma ^{-2}_{e_{1}}+{\mathbf {I}}_m\sigma ^{11}_{\upbeta } & \ldots & {\mathbf {I}}_m\sigma ^{1K}_{\upbeta }\\ \vdots & \ddots & \vdots & \vdots & \ddots & \vdots \\ {\mathbf {0}} & \ldots & {\mathbf {Z}}^{\prime}_{K}{\mathbf {X}}^{\prime}_{K}\sigma ^{-2}_{e_{K}} & {\mathbf {I}}_m\sigma ^{K1}_{\upbeta } & \ldots & {\mathbf {Z}}^{\prime}_{K}{\mathbf {Z}}_{K}\sigma ^{-2}_{e_{K}}+{\mathbf {I}}_m\sigma ^{KK}_{\upbeta }\\ \end{bmatrix} \begin{bmatrix} \hat{{\mathbf {b}}}_{1}\\ \vdots \\ \hat{{\mathbf {b}}}_{k}\\ \hat{\varvec{\upbeta }}_{1}\\ \vdots \\ \hat{\varvec{\upbeta }}_{K}\\ \end{bmatrix} = \begin{bmatrix} \sigma ^{-2}_{e_{1}}{\mathbf {X}}^{\prime}_{1}{\mathbf {y}}_{1}\\ \vdots \\ \sigma ^{-2}_{e_{K}}{\mathbf {X}}^{\prime}_{k}{\mathbf {y}}_{K}\\ \sigma ^{-2}_{e_{1}}{\mathbf {Z}}^{\prime}_{1}{\mathbf {y}}_{1}\\ \vdots \\ \sigma ^{-2}_{e_{K}}{\mathbf {Z}}^{\prime}_{K}{\mathbf {y}}_{K} \end{bmatrix},$$

where $\sigma ^{ij}_{\upbeta }$ is the element at position *ij* of $\varvec{\Sigma }^{-1}_{\upbeta }$.

The iterative Gauss–Seidel method with residual updates, as presented in [15], was used to solve the mixed-model equations without setting them up explicitly, while updating variances and covariances in each iteration. We define $\hat{{\mathbf {e}}} = [\hat{{\mathbf {e}}}_1~\hat{{\mathbf {e}}}_2~\ldots ~\hat{{\mathbf {e}}}_K]$ to be the vector of estimated residuals, which was initialized as $\hat{{\mathbf {e}}}^{(0)} = [{\mathbf {y}}^{\prime}_1~{\mathbf {y}}^{\prime}_2~\ldots ~{\mathbf {y}}^{\prime}_K]$. The estimated fixed effect *j* of environment *k* was updated in iteration *t* by:

$${\hat{b}}^{(t+1)}_{jk} = \frac{{\mathbf {x}}^{\prime}_{jk}\hat{{\mathbf {e}}}_k}{ {\mathbf {x}}^{\prime}_{jk}{\mathbf {x}}_{jk}},$$

and before moving to the next fixed effect, the residual vector was updated by:

$$\hat{{\mathbf {e}}}^{(new)}_k = \hat{{\mathbf {e}}}^{(old)}_k - {\mathbf {x}}_{jk}{\hat{b}}^{(t+1)}_{jk}.$$

For updating estimated marker effects, $\hat{\dot{\varvec{\upbeta }}}^{^{\prime}(t)}_j = [{\hat{\upbeta }}^{(t)}_{j1}~{\hat{\upbeta }}^{(t)}_{j2}~\ldots ~{\hat{\upbeta }}^{(t)}_{jK}]$ is defined as the vector of estimated marker effects for marker *j* and all *K* environments in iteration *t*, $\dot{{\mathbf {Z}}}_j = \oplus ^{K}_{k=1} {\mathbf {z}}_{jk}$ as a matrix containing scores for marker *j*, ${\mathbf {z}}_{jk}$ as an $n_k$ column vector for scores at marker *j* and environment *k*, and $\hat{\varvec{\Sigma }}^{(t)}_{e} = Diag\{{\hat{\sigma }}^{2(t)}_{e_1},{\hat{\sigma }}^{2(t)}_{e_2},~\ldots ~,{\hat{\sigma }}^{2(t)}_{e_K}\}$ as a diagonal matrix of estimated residual variances from all environments. Estimates of effects for marker *j* were initialized to zero and updated by:

2 $$\hat{\dot{\varvec{\upbeta }}}^{(t+1)}_j = \left( \hat{\varvec{\Sigma }}^{-1(t)}_{e}\dot{{\mathbf {Z}}}^{\prime}_j\dot{{\mathbf {Z}}}_j + \hat{\varvec{\Sigma }}^{-1(t)}_{\upbeta }\right) ^{-1} \hat{\varvec{\Sigma }}^{-1(t)}_{e}\dot{{\mathbf {Z}}}^{\prime}_j \left( \dot{{\mathbf {Z}}}_j\hat{\dot{\varvec{\upbeta }}}^{(t)}_j+\hat{{\mathbf {e}}}\right) ,$$

and before moving to the next marker, the residual vector is updated as:

$$\hat{{\mathbf {e}}}^{(new)} = \hat{{\mathbf {e}}}^{(old)} - \dot{{\mathbf {Z}}}^{\prime}_j\left( \hat{\dot{\varvec{\upbeta }}}^{(t+1)}_j - \hat{\dot{\varvec{\upbeta }}}^{(t)}_j\right) .$$

The term $\hat{\varvec{\Sigma }}^{-1(t)}_{e}\dot{{\mathbf {Z}}}^{\prime}_j\dot{{\mathbf {Z}}}_j$ of Eq. (2) is a $K\times K$ diagonal matrix with elements $\{{\hat{\sigma }}^{-2(t)}_{e1}{\mathbf {z}}^{\prime}_{j1} {\mathbf {z}}_{j1}, \ldots , {\hat{\sigma }}^{-2(t)}_{eK}{\mathbf {z}}^{\prime}_{jK} {\mathbf {z}}_{jK}\}$, and the term $\hat{\varvec{\Sigma }}^{-1(t)}_{e}\dot{{\mathbf {Z}}}^{\prime}_j(\dot{{\mathbf {Z}}}_j\hat{\dot{\varvec{\upbeta }}}^{(t)}_j+\hat{{\mathbf {e}}})$ can be computed as a vector of length *K* with elements $[ {\hat{\sigma }}^{-2(t)}_{e_1}({\mathbf {z}}^{\prime}_{j1} {\mathbf {z}}_{j1} {\hat{\upbeta }}^{(t)}_{j1} + {\mathbf {z}}_{j1}^{\prime} \hat{{\mathbf {e}}}_1), \ldots , {\hat{\sigma }}^{-2(t)}_{e_K}({\mathbf {z}}^{\prime}_{jK} {\mathbf {z}}_{jK} {\hat{\upbeta }}^{(t)}_{jK} + {\mathbf {z}}_{jK}^{\prime} \hat{{\mathbf {e}}}_K)]$. Values of ${\mathbf {z}}^{\prime}_{jk} {\mathbf {z}}_{jk}$ were calculated before iterations start for all combinations of markers (*j*) and environments (*k*).

To increase convergence rate, the order in which the marker effects are updated was randomized in each iteration. This approach is referred to as randomized Gauss–Seidel [8, 9].

### Solving variances and covariances

Genetic variances and covariances were updated by using the method proposed by either [12] or [13], called *Tilde-Hat* (TH) and *Pseudo Expectation* (PE), respectively. Both methods use the quadratic form $\tilde{\varvec{\upbeta }}^{\prime(t)}_k\hat{\varvec{\upbeta }}^{(t)}_k$, where $\hat{\varvec{\upbeta }}^{(t)}_k$ contains all estimated marker effects for environment *k* in iteration *t*, and:

3 $$\tilde{\varvec{\upbeta }}^{(t)}_k = {\mathbf {D}}^{-1(t)}_k{\mathbf {Z}}^{\prime}_k {\mathbf {M}}_{k} {\mathbf {y}}_k.$$

The two methods differ in matrix ${\mathbf {D}}^{-1(t)}_k$. In PE, ${\mathbf {D}}^{(t)}_k = {\mathbf {I}}_m$, whereas in TH,

4 $${\mathbf {D}}^{(t)}_k = Diag\{{\mathbf {Z}}_k^{\prime}{\mathbf {M}}_k {\mathbf {Z}}_k{\hat{\sigma }}^{-2(t)}_{e_k}+{\mathbf {I}}_m{\hat{\sigma }}^{kk(t)}_{\upbeta }\},$$

which denotes a diagonal matrix, and ${\mathbf {M}}_{k}={\mathbf {I}}_k-{\mathbf {X}}_{k} ( {\mathbf {X}}_{k}^{\prime}{\mathbf {X}}_{k})^{-1} {\mathbf {X}}^{\prime}_{k}$. As ${\mathbf {D}}^{(t)}_k$ is diagonal, ${\mathbf {M}}_{k}$ does not have to be explicitly generated, but only the diagonal of ${\mathbf {Z}}^{\prime}_{k} {\mathbf {M}}_{k} {\mathbf {Z}}_{k}$ needs to be computed once before iterations start and stored. This computation can be done efficiently, as shown in Appendix 1. When the intercept is the only fixed effect, and both ${\mathbf {y}}_k$ and the columns of ${\mathbf {Z}}_k$ are centered, ${\mathbf {M}}_k$ can be omitted.

Estimates of genetic and residual variances for environment *k* were initialized to ${\hat{\sigma }}^{2(0)}_{\upbeta _k} = 0.5\cdot \sigma ^2_{y_k}/(m\cdot \overline{\sigma ^2}_{Z_k}$) and ${\hat{\sigma }}^{2(0)}_{e_k} = 0.5\cdot \sigma ^2_{y_k}$, respectively, where $\sigma ^2_{y_k}$ is the sample variance of phenotypes and $\overline{\sigma ^2}_{Z_k} = \frac{1}{m}\sum ^m_{j=1}\sigma ^2_{Z_{k_j}}$ is the average of marker-score variances across the *m* columns of ${\mathbf {Z}}_k$. Estimates of genetic covariances were initialized to zero. The estimate of variance of marker effects for environment *k* was updated by:

5 $${\hat{\sigma }}^{2(t+1)}_{\upbeta _k} = \frac{\tilde{\varvec{\upbeta }}^{^{\prime}(t)}_k\hat{\varvec{\upbeta }}^{(t)}_k}{tr\left( {\mathbf {D}}^{-1(t)}_k {\mathbf {Z}}^{\prime}_k {\mathbf {M}}_{k} {\mathbf {Z}}_{k}\right) },$$

where ${\mathbf {Z}}_k$ contains marker scores for environment *k*, $tr(\cdot )$ is the trace operator, and $tr({\mathbf {D}}^{-1(t)}_k {\mathbf {Z}}^{\prime}_k {\mathbf {M}}_{k} {\mathbf {Z}}_k)$ is the expected value of $\tilde{\varvec{\upbeta }}^{\prime(t)}_k\hat{\varvec{\upbeta }}^{(t)}_k$, as derived in [12] and in Appendix 2. The estimate of the covariance between environments *k* and $k^{\prime}$ was updated by:

6 $${\hat{\sigma }}^{(t+1)}_{\upbeta _{kk^{\prime}}} = \frac{\tilde{\varvec{\upbeta }}^{^{\prime}(t)}_k\hat{\varvec{\upbeta }}^{(t)}_{k^{\prime}} + \tilde{\varvec{\upbeta }}^{^{\prime}(t)}_{k^{\prime}}\hat{\varvec{\upbeta }}^{(t)}_{k}}{tr\left( {\mathbf {D}}^{-1(t)}_{k} {\mathbf {Z}}^{\prime}_{k} {\mathbf {M}}_{k} {\mathbf {Z}}_{k}\right) +tr({\mathbf {D}}^{-1(t)}_{k^{\prime}} {\mathbf {Z}}^{\prime}_{k^{\prime}} {\mathbf {M}}_{k^{\prime}} {\mathbf {Z}}_{k^{\prime}})},$$

as proposed by [13] and derived in Additional file 1, and residual variances were updated by

7 $${\hat{\sigma }}^{2(t+1)}_{e_k} = \frac{\left( {\mathbf {M}}_{k}{\mathbf {y}}_{k}\right) ^{\prime}\hat{{\mathbf {e}}}_k}{n_{k}-r_{k}}$$

as in [15], where $r_k$ is the number of linear independent columns of ${\mathbf {X}}_k$.

Bending of ${\hat{\Sigma }}_{\upbeta }$ as described in [16] was used after an iteration when it was not positive definite. The iterative scheme was repeated until a mean-squared convergence of $10^{-8}$ was reached for effects, variances, and covariances. The combination of the randomized Gauss–Seidel solver with either of the two methods for variance component estimation, i.e., TH or PE, is referred to here as THGS and PEGS, respectively. An implementation of PEGS is provided in the R package bWGR (2.0) [17], function mrr.

### Exact THGS

For balanced experimental designs, when the intercept is the only fixed effect, and either a principal components [18] or eigenvector regression [19–21] is used, THGS is exact. This is demonstrated in Appendix 3. By either using a singular-value decomposition of ${\mathbf {Z}}_k$ or an eigenvalue decomposition (EVD) of ${\mathbf {Z}}^{\prime}_{k}{\mathbf {Z}}_{k}$, a matrix of eigenvectors, ${\mathbf {U}}_{k}$, and a diagonal matrix of eigenvalues, $\varvec{\Lambda }_k$, can be calculated. By fitting $\check{{\mathbf {Z}}}_k = {\mathbf {Z}}_{k}{\mathbf {U}}_k$ rather than ${\mathbf {Z}}_{k}$ in model (1), ${\mathbf {Z}}^{\prime}_{k}{\mathbf {M}}_k{\mathbf {Z}}_k$ in Eq. (4) becomes a diagonal matrix of eigenvalues, $\varvec{\Lambda }_k$. Thus, ${\mathbf {D}}^{(t)}_k$ in Eqs. (5) and (6) can be written as:

8 $${\mathbf {D}}^{(t)}_{k} = \varvec{\Lambda }_{k}{\hat{\sigma }}^{-2(t)}_{e_{k}}+{\mathbf {I}}_{m}{\hat{\sigma }}^{kk(t)}_{\upbeta }.$$

This does not apply to PEGS, because it uses ${\mathbf {D}}^{(t)}_{k} = {\mathbf {I}}_{m}$.

### Alternative methods

As a gold standard for low biases and standard errors of both GEBV and variance components, empirical genomic best linear unbiased predictions (GBLUP) [22] were obtained by REML [23] for balanced experimental designs as follows. The genomic relationship matrix (${\mathbf {G}}$) was diagonalized and the statistical model was transformed by the eigenvectors of an eigenvalue decomposition of ${\mathbf {G}}$ [7] (see Appendix 4). Eigenvectors of the smallest eigenvalues, which explained the last 1% of the variation in ${\mathbf {G}}$ were neglected [24]. The transformed model was fitted using ASREML-R [25]. For unbalanced experimental designs, ASREML 4.2, AIREMLF90 or REMLF90 did not return results for the full multivariate models in this simulation study. Thus, to obtain an upper bound of accuracy of GEBV, GBLUP were calculated using the true simulated variance components. This method was called true value Gauss–Seidel (TVGS).

Runtimes of the proposed and other methods were compared only for balanced designs. In addition to the REML approach described above, ${\mathbf {G}}$ was used in its natural, dense form and 0.01 was added to its diagonal to render it positive definite. The expectation maximization (EM) REML algorithm of REMLF90 [26] and the average information (AI) REML algorithms of ASREML 4.2 [23, 25] and AIREMLF90 [27] were used with their options for dense equations operations *!gdense* and *use_yams*, respectively. In addition, the Gibbs sampler of GIBBSF90 was run for comparison.

Univariate THGS (UV-THGS), which analyzes phenotypes of only one environment at a time with the randomised Gauss–Seidel solver and TH, was run to evaluate the increase in accuracy of GEBV with multivariate THGS over univariate THGS. Table 1 summarizes the methods used in this study.

**Table 1:** Summary of methods used in the simulations

	TVGS	PEGS	THGS	UV-THGS	REML
Effect type in the model	Marker	Marker	Marker	Marker	Polygenic
Multivariate	Yes	Yes	Yes	No	Yes
(Co)variance estimation^a^	True values	PE	TH	TH	REML
Orthogonalization	No	No	No	No	Yes

^a^*PE* pseudo expectation, *TH* tilde-hat, *REML* restricted maximum likelihood, *TVGS* true-value Gauss–Seidel, *PEGS* pseudo expectation Gauss–Seidel, *THGS* tilde-hat Gauss–Seidel, *UV-THGS* univariate-tilde-hat Gauss–Seidel

### Data and evaluation statistics

Phenotypic data for five scenarios were simulated to evaluate bias and accuracy of GEBV within environments, runtime, and biases and standard errors of estimates of heritabilities and genetic correlations (Table 2). The genotypes used in the simulations came from a wheat [28–31] and a soybean dataset [21, 32, 33], which have been used in multiple genomic prediction studies, and are available through the R packages BGLR and SoyNAM, respectively.

**Table 2:** Summary of simulated scenarios

	Scenario 1	Scenario 2	Scenario 3	Scenario 4	Scenario 5
Number of environments (traits)	10	10	10	10–400	10–400
Number of environments per line	10	1	0–10	0–400	0–400
Number of lines per environment	599	514	250-3000	4628	4628
% of lines per environment	100%	10%	5-60%	90%	90%
Number of phenotypic records	5990	51,420	30,000	1,851,120	1,851,120
Number of markers	1279	4311	4311	4311	42,034
Species	Wheat	Soy	Soy	Soy	Soy

**Scenario 1** contained simulated phenotypes from inbred lines that were grown in the same ten environments, using 599 inbred lines from CIMMYT’s Global Wheat Program [28, 29] that were genotyped at 1279 DArT markers [34]. **Scenario 2** contained simulated phenotypes from different inbred lines grown in ten different environments, using 5142 recombinant inbred lines from the SoyNAM project [35, 36] genotyped for 4311 single nucleotide polymorphism (SNP) markers. These lines were randomly allocated to ten different environments, and each line was observed in only one environment. **Scenario 3** was used to study the evaluation statistics for an increasing number of soy inbred lines in each of the ten environments. Thus, each line could be present in multiple environments. **Scenario 4** was used to study runtime of PEGS and THGS for an increasing number of environments (response variables), i.e., 10, 50, 100, 200 and 400, using the SoyNAM dataset with a random 10% of lines missing in each environment. **Scenario 5** was used to study runtime with a higher marker density, using the SoyNAM dataset and genotyped at 42,034 SNPs that were obtained from the original SNPs plus from a linkage disequilibrium-based imputation of SNPs, as described in [36].

Phenotypes were simulated by summing true genomic breeding values (TBV) and residuals. TBV for environment *k* were sampled as ${\mathbf {Z}}\varvec{\upbeta }_k$, where ${\mathbf {Z}}$ contains marker scores of inbred lines from all environments and the true marker effects in $\varvec{\upbeta }_k$ were taken from $\varvec{\upbeta }^{\prime} = [\varvec{\upbeta }^{\prime}_1 ~ \varvec{\upbeta }^{\prime}_2 ~ \ldots \varvec{\upbeta }^{\prime}_K]$, which was sampled from $N({\mathbf {0}}, \varvec{\Sigma }_{\upbeta }\otimes {\mathbf {I}}_m$), where $\varvec{\Sigma }_{\upbeta } = \alpha ^{-1}\varvec{\Sigma }_{g}$, $\alpha = \sum ^J_{j=1}{\sigma ^2_{Z_j}}$, $\sigma ^2_{Z_j}$ is the variance of marker scores in ${\mathbf {Z}}$ at marker *j*, and $\varvec{\Sigma }_{g}$ is the additive genetic variance-covariance matrix with 1 on the diagonal and genetic correlations on the off-diagonals. Residuals were sampled from $N(0,(1-h^2)h^{-2})$, where $h^2$ is the heritability in an environment. Three heritabilities (0.2, 0.5, and 0.8) and three ranges of genetic correlations, low (0.2–0.4), medium (0.4––0.6), and high (0.6–0.8) were considered. Correlations were sampled from a uniform distribution within each range. Each simulation scenario was replicated 100 times.

Biases and standard errors of estimates of heritabilities and genetic correlations were calculated as the average and standard deviation, respectively, of estimated minus true simulated values across replicates. GEBV for environment *k* were calculated as ${\mathbf {Z}}_k\hat{\varvec{\upbeta }}_k$, and bias and accuracy of these GEBV were calculated as the regression coefficient of TBV on GEBV and as the correlation between TBV and GEBV, respectively.

## Results

### Runtime

The average runtimes for the different methods used in scenario 1 are presented in Table 3. Multivariate PEGS and THGS took 0.4 and 0.3 s, respectively, univariate THGS aggregated across ten environments 0.2 s, and AI-REML using ASREML-R 3.3 s when the genomic relationship matrix was diagonalized by eigenvalue decomposition. Standard implementations of REML based on the dense genomic relationship matrix ranged from 109.8 to 1250.7 s, whereas the Gibbs sampler took 559.8 s.

**Table 3:** Average runtime in seconds (s.e.) for the balanced experimental design in scenario 1 based on 100 replicates of the simulation

Method^a^	Software	Model^c^	Runtime
PEGS	–	RR	0.4 (0.0)
THGS	–	RR	0.3 (0.0)
UV-THGS	–	RR	0.2 (0.0)
AI-REML (EVD)	ASREML-R	GBLUP	3.3 (0.3)
AI-REML	ASREML 4.2	GBLUP	272.6 (36.5)
AI-REML	AIREMLF90	GBLUP	109.8 (2.4)
EM-REML	REMLF90	GBLUP	1250.7 (11.7)
Gibbs sampling^b^	GIBBS3F90	GBLUP	559.8 (9.6)

^a^*PEGS* pseudo expectation Gauss–Seidel, *THGS* tilde-hat Gauss–Seidel, *UV-THGS* univariate-tilde-hat Gauss–Seidel, *AI* average information, *REML* restricted maximum likelihood, *EVD* eigenvalue decomposition, *EM* expectation maximization

^b^10,000 MCMC iterations

^c^*RR* ridge-regression, *GBLUP* genomic best linear unbiased prediction

**Fig. 1:**
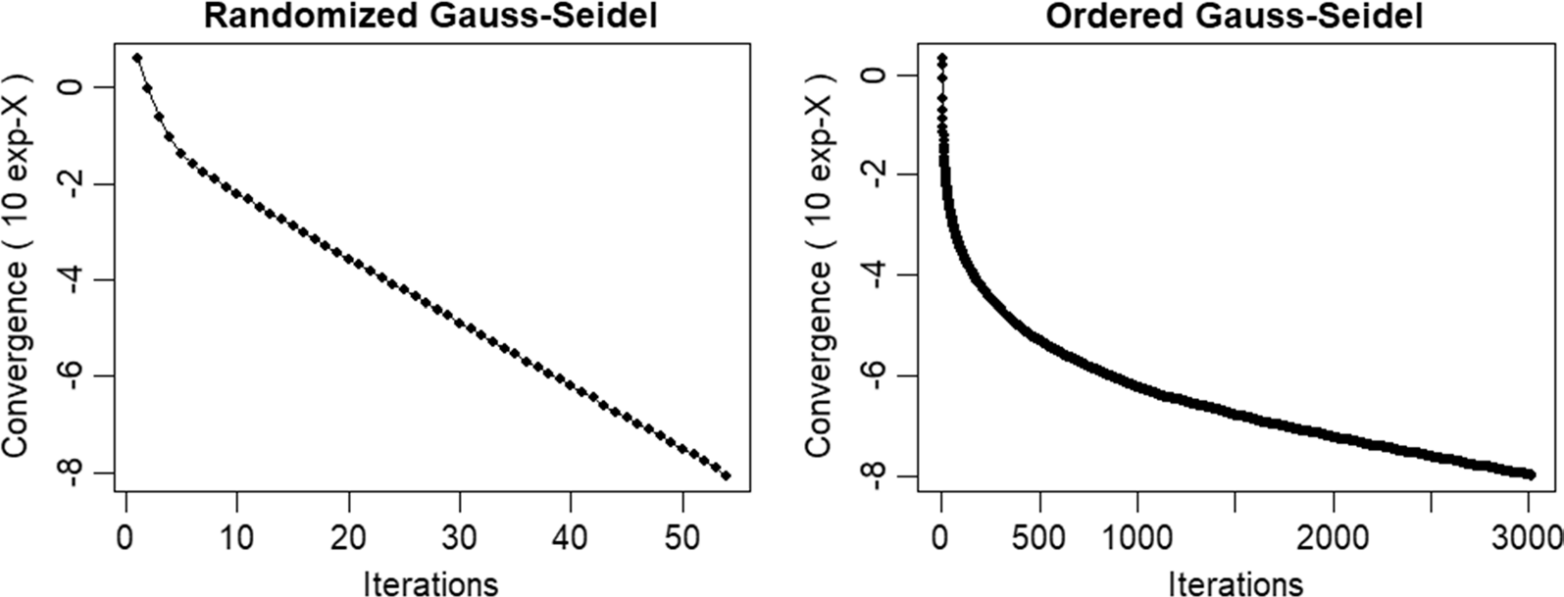
Convergence of the Gauss–Seidel solver with (left) and without (right) randomizing the order in which marker effects were updated for one replicate of the simulation of scenario 2

Figure 1 shows convergence of the Gauss–Seidel solver with and without randomizing the order in which marker effects were updated for one replicate of scenario 2. The algorithm converged after 54 iterations with randomization, but required more than 3000 iterations without randomization.

Table 4 depicts average runtime in minutes for PEGS, THGS, and UV-THGS with and without randomizing the marker order in the Gauss–Seidel solver, as well as an increasing number of environments (scenario 4) and markers (scenario 5). The runtimes of PEGS and THGS were similar, and randomizing the marker order shortened runtimes. Without randomization, the multivariate models that fitted 42,034 SNPs did not converge within 2000 iterations. Runtimes of PEGS and THGS increased exponentially with the number of environments from 0.2 min for ten environments to 448 min for 400 environments when using 4311 SNPs. Runtime of UV-THGS, in contrast, increased linearly from 0.1 to 4.3 min under the same conditions. With randomization, runtime increased with an increasing number of markers, from 0.2 min for 4311 SNPs to 0.8 min for 42,034 SNPs and ten environments, and from 80.5 to 123.2 min for 200 environments. Without randomization, runtime increased to 3057.3 min for 42,034 SNPs and 200 environments.

**Table 4:** Average runtime in minutes (s.e.) of the Gauss–Seidel solver with and without randomizing the order of markers for updating marker effects, with increasing numbers of SNPs and environments, based on 10 replicates of scenarios 4 (4311 SNPs) and 5 (42,034 SNPs)

Randomized	Number of SNPs	Number of environments	PEGS	THGS	UV-THGS
Yes	4311	10	0.2 (0)	0.2 (0)	0.1 (0)
Yes	4311	50	3.5 (0.4)	3.5 (0.4)	0.6 (0)
Yes	4311	100	14.4 (2)	14.4 (1.8)	1.1 (0)
Yes	4311	200	80.5 (10.1)	79.2 (11)	2.3 (0.1)
Yes	4311	400	459.3 (55.1)	448 (58)	4.3 (0.1)
No	4311	10	5.5 (1)	5.4 (0.9)	1.9 (0.2)
No	4311	50	44.9 (7)	44.6 (6.9)	9.3 (1.1)
No	4311	100	120.9 (10.1)	123.7 (9.9)	20 (1.8)
No	4311	200	361.1 (48.9)	364.6 (44.4)	39.3 (2.8)
No	4311	400	1261.8 (115.8)	1261.7 (107.9)	74.1 (8.3)
Yes	42,034	10	0.8 (0.1)	0.8 (0)	1.2 (0.1)
Yes	42,034	50	9.9 (0.4)	12.5 (1.3)	5.7 (0.4)
Yes	42,034	100	36.4 (1.4)	29.2 (2.7)	11.3 (0.6)
Yes	42,034	200	123.2 (17.1)	119.7 (10.1)	22.5 (2)
Yes		400	730 (64.4)	802.2 (118.2)	46.4 (4.1)
No	42,034	10	64^a^ (14.7)	64.2^a^ (16)	14.5 (5.1)
No	42,034	50	540.2^a^ (38.3)	536^a^ (26.8)	106.5 (63.2)
No	42,034	100	1109.6^a^ (71.5)	1148.1^a^ (109.3)	181.4 (40.6)
No	42,034	200	3057.3^a^ (292.7)	3001.2^a^ (259)	310.3 (114.8)

*PEGS* pseudo expectation Gauss–Seidel, *THGS* tilde-hat Gauss–Seidel, *UV-THGS* univariate-tilde-hat Gauss–Seidel

^a^Did not converge within 2000 iterations

### Accuracy and bias of GEBV

**Fig. 2:**
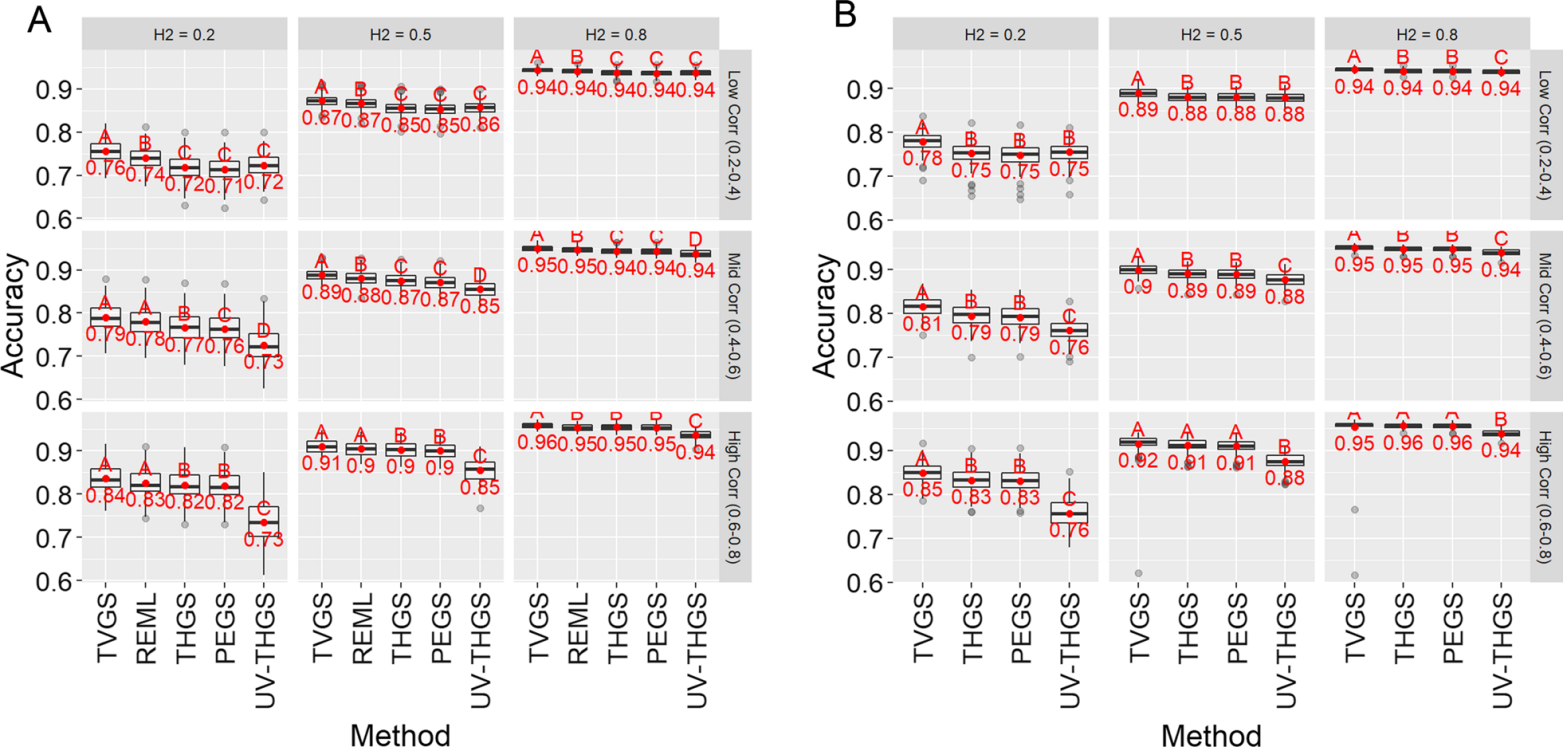
Accuracy of GEBV for scenario 1 (**a** wheat dataset) and scenario 2 (**b** soybean dataset) for different true heritabilities (columns) and genetic correlations (rows), based on 100 replicates of the simulation. Letters indicate Tukey’s test of multiple comparisons ($\alpha =0.05$)

Accuracy of GEBV increased with increasing heritability and genetic correlation, as expected (Fig. 2). It was 0.03 to 0.09 higher for multivariate approaches than for univariate THGS when heritability was low and the genetic correlation was medium to high (Fig. 2a, b, lower left panels). For most genetic parameters for scenario 1, REML provided a 0.01 higher accuracy than PEGS and THGS. For low heritability and low genetic correlations, however, REML resulted in a 0.02 higher accuracy and UV-THGS was as accurate as PEGS and THGS (Fig. 2a, upper left panel). The latter was also true for scenario 2. After additional simulations of scenario 1 for low heritability and low genetic correlations, accuracies of PEGS and THGS became larger than those of UV-GS and approached those of REML with increasing number of environments (Additional file 2). Even REML tended to have lower accuracies for low heritability and low genetic correlation than TVGS (Fig. 2a, upper left panel). Differences for TVGS with both PEGS and THGS were similar for scenarios 1 and 2 (Fig. 2a, b). PEGS and THGS were not significantly different for scenarios 1 and 2.

**Fig. 3:**
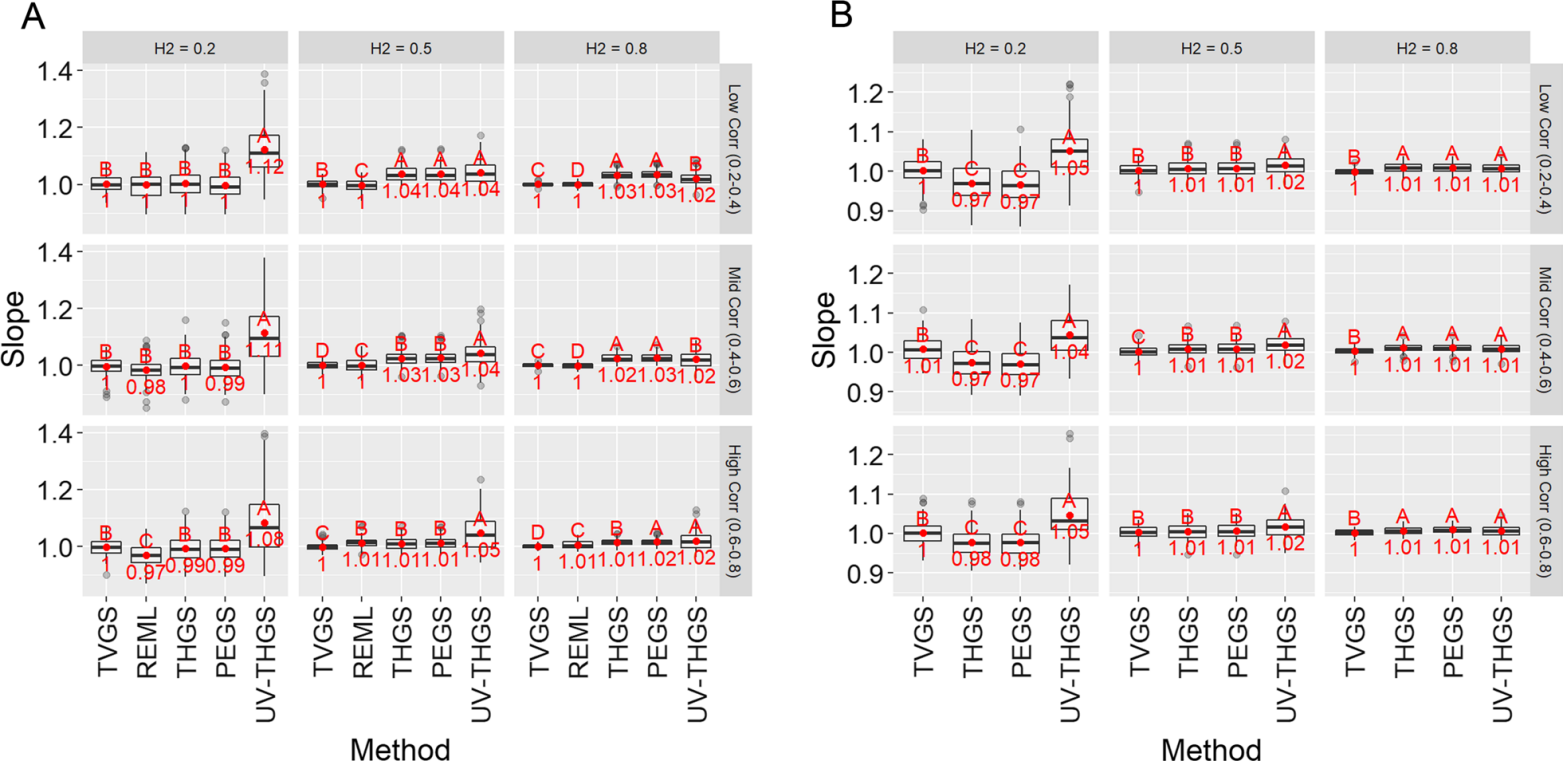
Regression of true breeding values on GEBV (slope) for scenario 1 (**a** wheat dataset) and scenario 2 (**b** soybean dataset) for different true heritabilities (columns) and genetic correlations (rows), based on 100 replicates of the simulation. Letters indicate Tukey’s test of multiple comparisons ($\alpha =0.05$)

Regression coefficients of TBV on GEBV are shown in Fig. 3. For scenario 1 and low heritability, they were 1 for PEGS and THGS, close to 1 for REML, and significantly above 1 for UV-THGS. This bias for UV-THGS decreased with increasing heritability. For medium to high heritabilities, however, PEGS and THGS slightly underestimated (values > 1) the TBV, while REML was usually unbiased, with a value of 1 (Fig. 3a). The bias for PEGS and THGS decreased with increasing genetic correlation. For scenario 2 (Fig. 3b), PEGS and THGS slightly overestimated TBV (values < 1) for low heritability, but slightly underestimated TBV (values > 1) for medium to high heritabilities.

### Bias and standard error of parameters

**Fig. 4:**
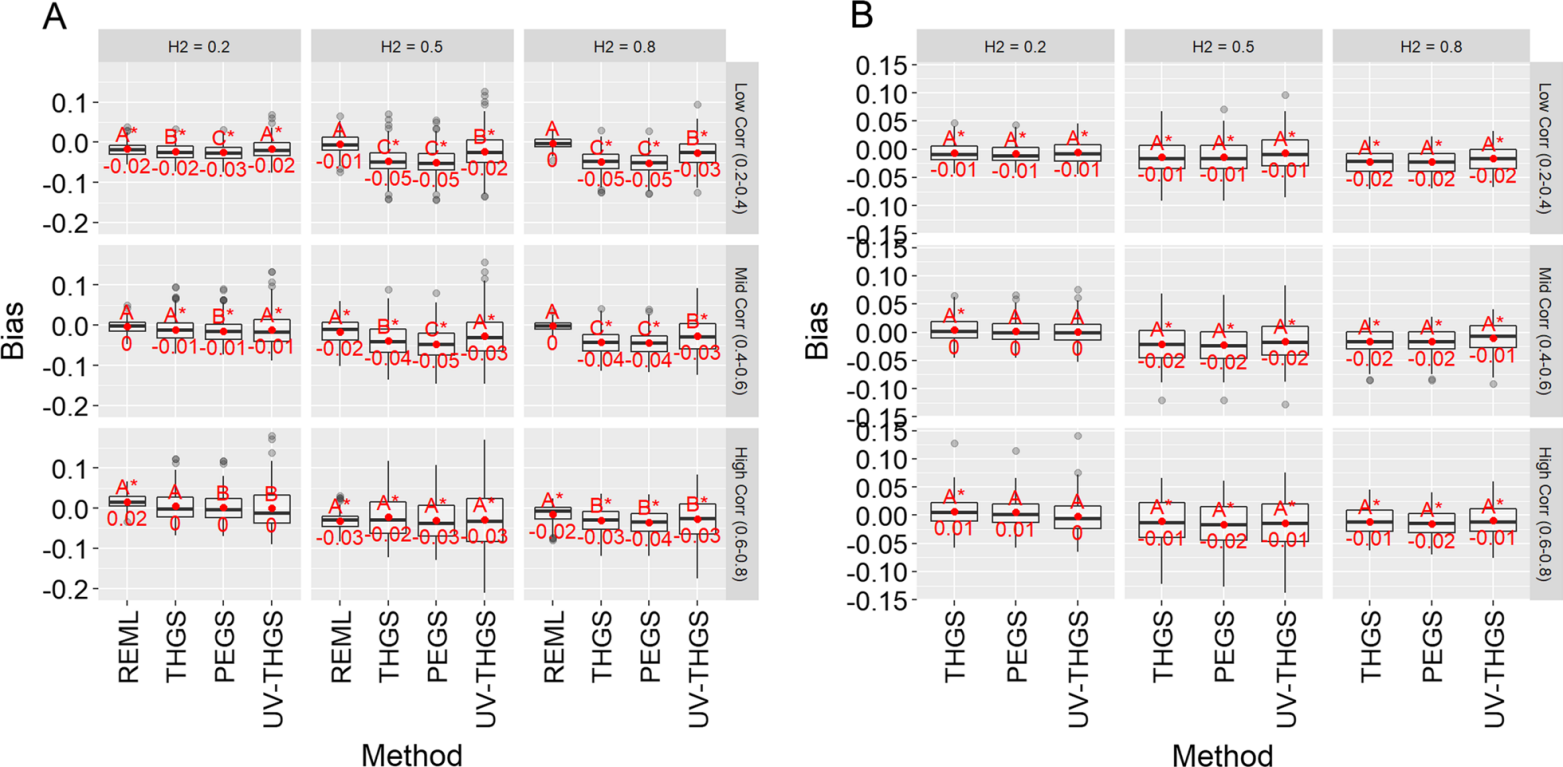
Bias of estimates of heritability for scenario 1 (**a** wheat dataset) and scenario 2 (**b** soybean dataset) for different true heritabilities (columns) and true genetic correlations (rows), based on 100 replicates of the simulation. Letters indicate Tukey’s test of multiple comparisons ($\alpha =0.05$). Asterisk indicates that the mean is significantly different from zero ($\alpha =0.05$)

Figure 4 shows the bias of estimates of heritabilities for scenarios 1 and 2 and different true genetic parameters. For both scenarios, estimates of heritabilities tended to be downward biased. For PEGS and THGS, the bias was smallest or even zero for low heritability and medium to high genetic correlations (Fig. 4, bottom left panels) and their biases decreased with increasing genetic correlations. The bias for UV-THGS tended to be lower than for PEGS and THGS. REML provided the least biased heritability estimates for scenario 1.

**Fig. 5:**
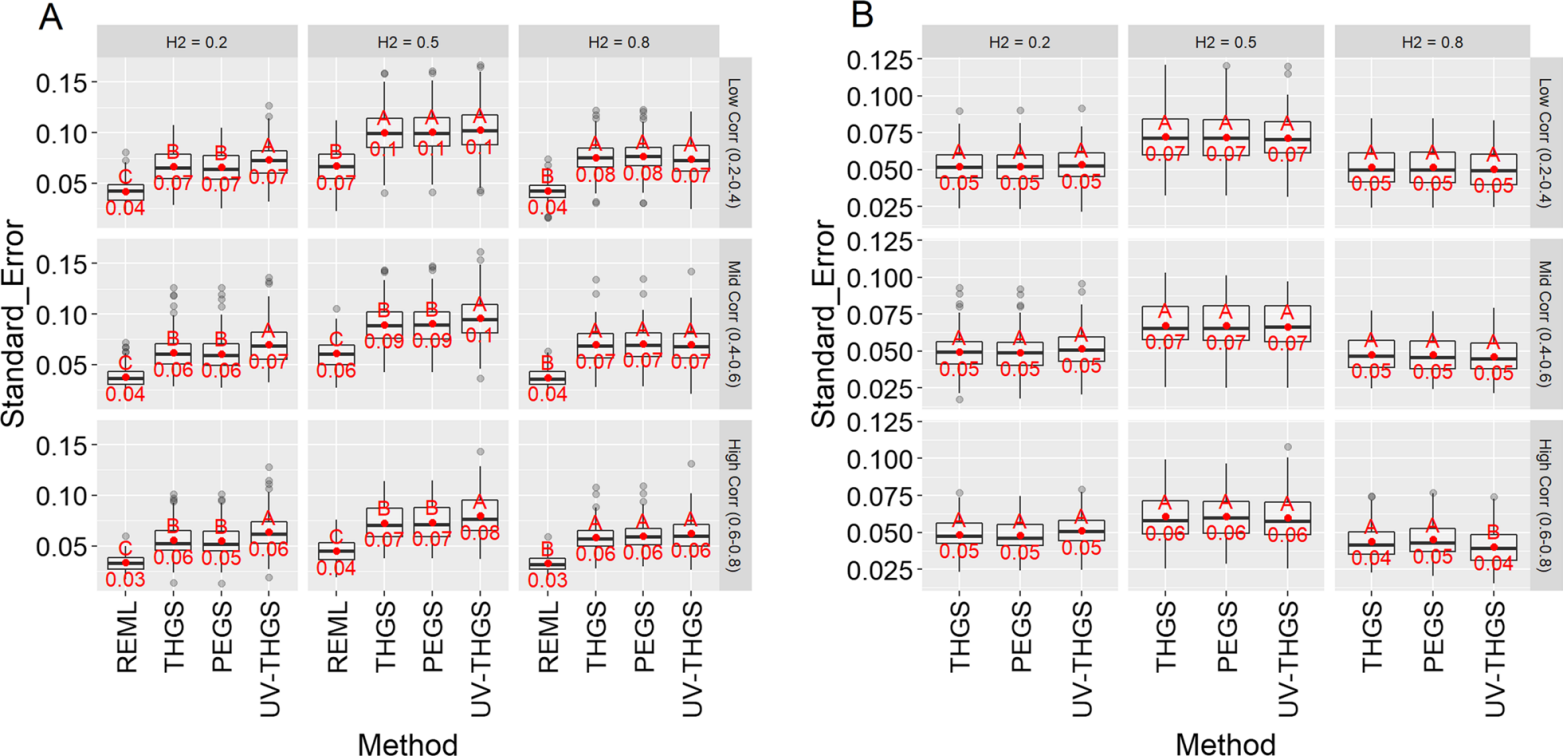
Standard error of estimates of heritability for scenario 1 (**a** wheat dataset) and scenario 2 (**b** soybean dataset) for different true heritabilities (columns) and true genetic correlations (rows), based on 100 replicates of the simulation. Letters indicate Tukey’s test of multiple comparisons ($\alpha =0.05$)

Figure 5 shows standard errors of estimates of heritabilities for scenarios 1 and 2 and different true genetic parameters. Standard errors were higher for scenario 1 than for scenario 2, higher for medium than for low and high heritabilities, highest for low genetic correlations, and decreased as the genetic correlation increased. Standard errors were 60 to 100% higher for PEGS and THGS than for REML.

**Fig. 6:**
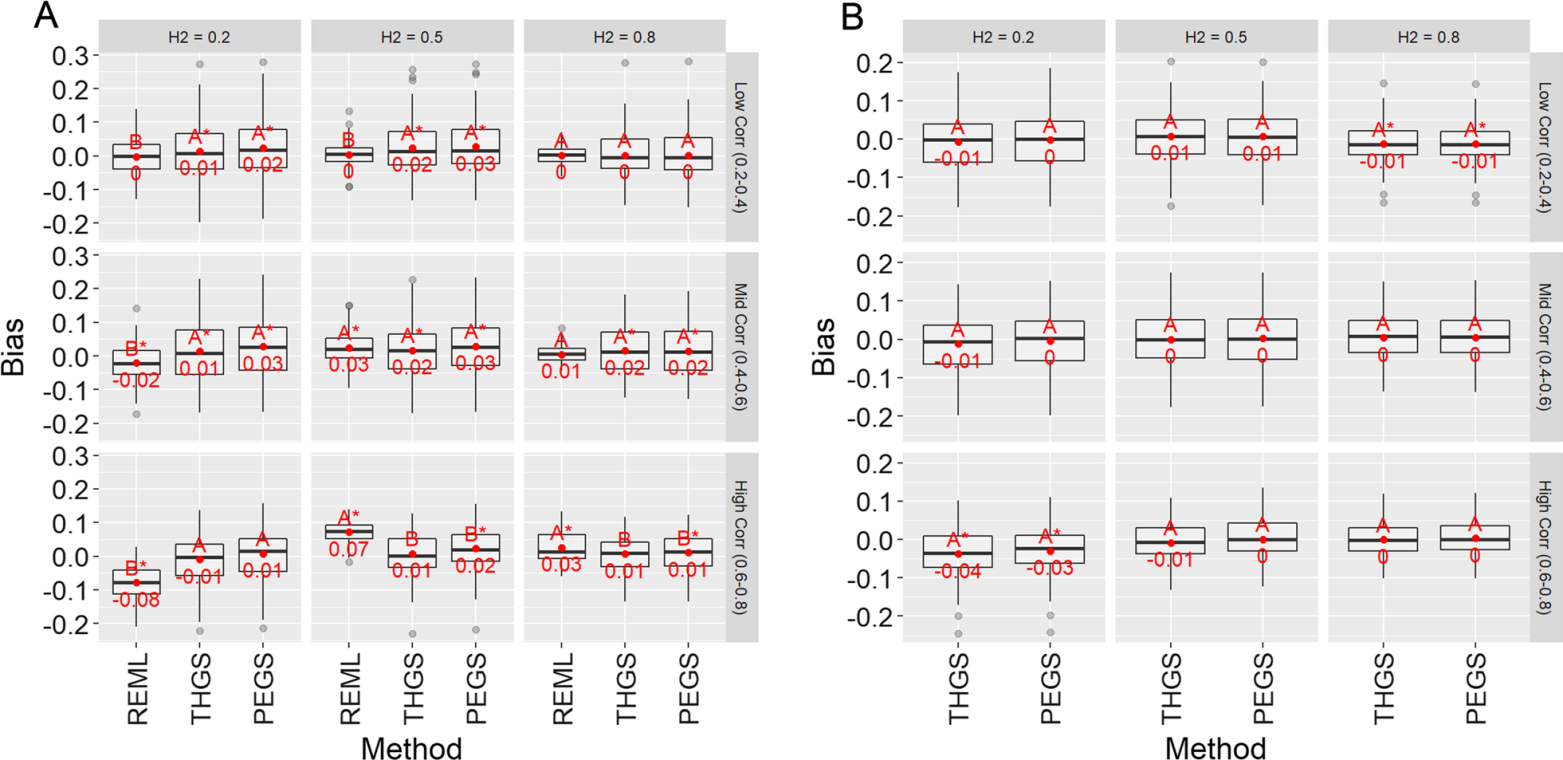
Bias of estimates of genetic correlation for scenario 1 (**a** wheat dataset) and scenario 2 (**b** soybean dataset) for different true heritabilities (columns) and true genetic correlations (rows), and based on 100 replicates of the simulation. Letters indicate Tukey’s test of multiple comparison ($\alpha =0.05$). Asterisk indicates that the mean is significantly different from zero ($\alpha =0.05$)

**Fig. 7:**
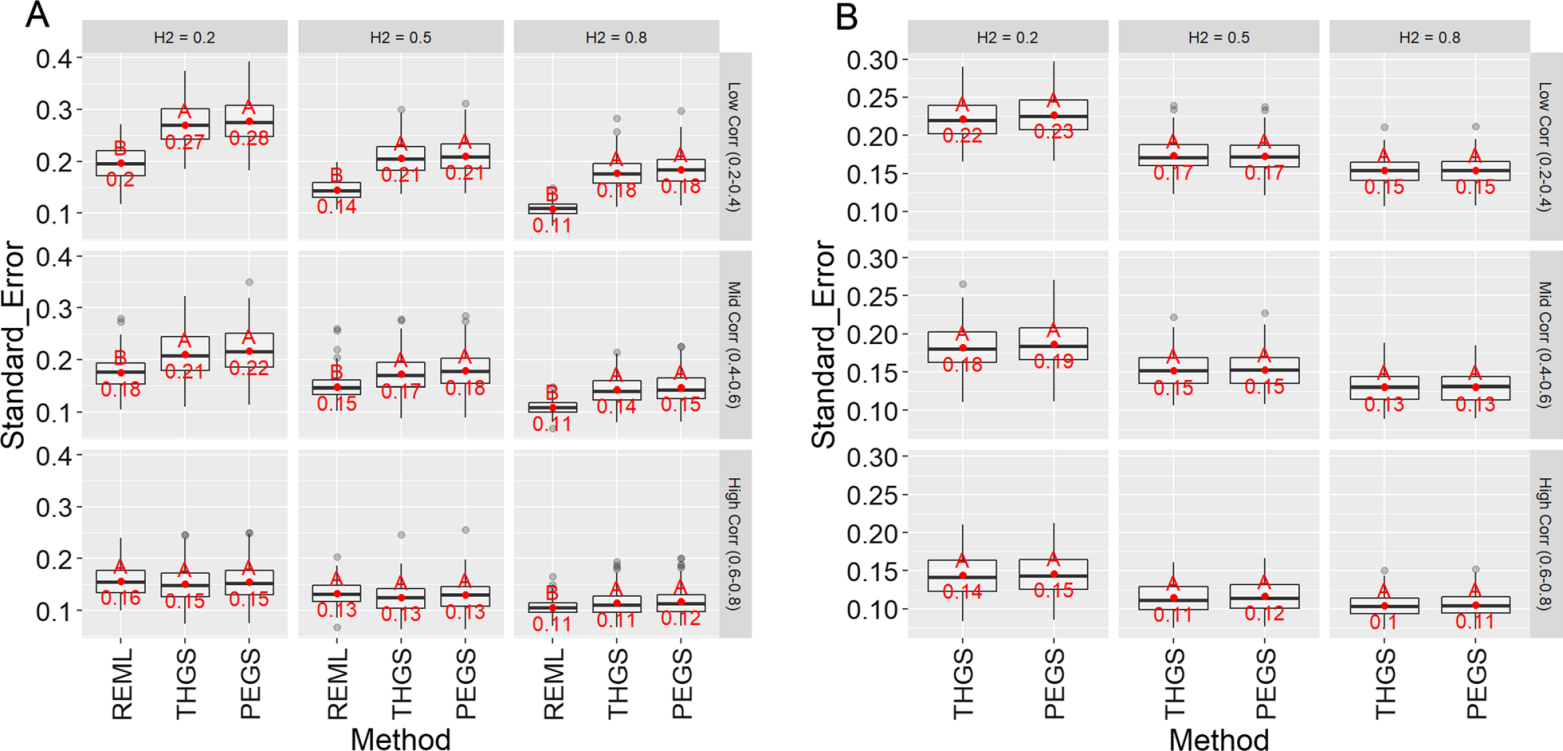
Standard error of estimates of genetic correlations for scenario 1 (**a** wheat dataset) and scenario 2 (**b** soybean dataset) for different true heritabilities (columns) and true genetic correlations (rows), based on 100 replicates of the simulation. Letters indicate Tukey’s test of multiple comparisons ($\alpha =0.05$). Asterisk indicates that the mean is significantly different from zero ($\alpha =0.05$)

Figures 6 and 7 show the bias and standard errors of estimates of genetic correlations for scenarios 1 and 2. Bias tended to be low for PEGS and THGS for scenario 2, except for low heritability and high genetic correlations (Fig. 6b, lower left panel). For scenario 1 and high genetic correlations (Fig. 6a, lower left panel), REML had large biases, with absolute values of up to 0.08, compared to 0.01 for THGS. For low and medium true genetic correlations and for scenario 2, REML and the proposed methods had similar biases, and they were not significantly different for PEGS and THGS. As standard software for REML did not return results for the full model and the unbalanced designs for scenario 2, bivariate models were ran and the resulting estimates of the genetic correlations are given in Additional file 3.

Standard errors of estimates of the genetic correlations decreased with increasing heritability and genetic correlations (Fig. 7). Standard errors were always similar for PEGS and THGS, but higher than for REML for low to medium genetic correlations. For high genetic correlations, standard errors were similar for all methods. Standard errors were lower for scenario 2 than for scenario 1.

As the number of observations per environment increased in scenario 3, standard errors of estimates of genetic parameters decreased, bias of estimates of genetic correlations decreased, but bias of estimates of heritabilities did not approach zero even with 3000 observations per environment (Table 5). Additional file 4 demonstrates the outcome when all 5142 lines were observed in all environments: heritabilities estimated with THGS were unbiased, and genetic correlations estimated with PEGS or THGS were unbiased.

**Table 5:** Accuracy of GEBV, regression of TBV on GEBV (Slope), and bias and standard error (SE) of estimates of heritabilities (${\hat{h}}^2$) and genetic correlations (GC) with increasing numbers of observations per environment (Obs/Env) in scenario 3, based on 100 replicates of the simulation

Method	Obs/Env	Accuracy	Slope	Bias of ${\hat{h}}^2$	SE of ${\hat{h}}^2$	Bias of GC	SE of GC
PEGS	250	0.82 (0.03)	0.98 (0.03)	− 0.01 (0.03)	0.07 (0.01)	− 0.01 (0.06)	0.17 (0.02)
PEGS	3000	0.96 (0.03)	1.00 (0.03)	− 0.01 (0.03)	0.04 (0.01)	0.00 (0.06)	0.13 (0.02)
THGS	250	0.82 (0.03)	0.98 (0.04)	0.00 (0.03)	0.07 (0.01)	− 0.02 (0.06)	0.17 (0.02)
THGS	3000	0.96 (0.03)	1.00 (0.03)	− 0.01 (0.03)	0.04 (0.01)	0.00 (0.06)	0.13 (0.02)
UV-THGS	250	0.79 (0.03)	1.04 (0.03)	− 0.01 (0.03)	0.07 (0.01)	–	–
UV-THGS	3000	0.95 (0.03)	1.00 (0.04)	− 0.01 (0.03)	0.04 (0.01)	–	–

Standard errors of statistics are in parenthesis

*PEGS* pseudo expectation Gauss–Seidel, *THGS* tilde-hat Gauss–Seidel, *UV-THGS* univariate-tilde-hat Gauss–Seidel

### Orthogonalization

Table 6 presents bias and accuracy of GEBV, as well as bias and standard errors of estimates of genetic parameters with and without using eigenvalue decomposition (EVD). THGS-EVD provided unbiased GEBV (Slope = 1) and its accuracy was 0.01 higher than for THGS and thus equal to the accuracy of REML. Estimates of the genetic correlations of THGS-EVD were unbiased and had lower standard errors than those obtained with THGS. The accuracy of GEBV from UV-THGS-EVD did not increase compared to that from UV-THGS, suggesting that the increase of accuracy for THGS-EVD resulted from a higher accuracy of estimates of genetic correlations. Biases and standard errors of estimates of genetic parameters, as well as biases and accuracies of GEBV were not different for PEGS and PEGS-EVD.

**Table 6:** Accuracy of GEBV, regression of TBV on GEBV (Slope), and bias and standard error (SE) of estimates of heritabilities (${\hat{h}}^2$) and genetic correlations (GC) with and without eigenvalue decomposition (EVD), based on 100 replicates of the simulation of scenario 1

Method	Accuracy	Slope	Bias of ${\hat{h}}^2$	SE of ${\hat{h}}^2$	Bias of GC	SE of GC
REML-EVD	0.87 (0.02)	1.00 (0.03)	− 0.01 (0.02)	0.04 (0.01)	0.00 (0.04)	0.14 (0.03)
PEGS	0.86 (0.02)	1.02 (0.03)	− 0.03 (0.04)	0.07 (0.02)	0.02 (0.08)	0.18 (0.04)
PEGS-EVD	0.86 (0.02)	1.02 (0.03)	− 0.04 (0.04)	0.07 (0.02)	0.02 (0.08)	0.18 (0.04)
THGS	0.86 (0.02)	1.02 (0.03)	− 0.03 (0.04)	0.07 (0.02)	0.01 (0.08)	0.17 (0.04)
THGS-EVD	0.87 (0.02)	1.00 (0.03)	− 0.02 (0.03)	0.05 (0.01)	0.00 (0.04)	0.13 (0.02)
UV-THGS	0.84 (0.04)	1.06 (0.09)	− 0.02 (0.05)	0.08 (0.02)	–	–
UV-THGS-EVD	0.84 (0.03)	1.03 (0.04)	− 0.03 (0.03)	0.05 (0.01)	–	–

*REML* restricted maximum likelihood, *EVD* eigenvalue decomposition, *PEGS* pseudo expectation Gauss–Seidel, *THGS* tilde-hat Gauss–Seidel, *UV-THGS* univariate-tilde-hat Gauss–Seidel

## Discussion

Our main goal was to develop an algorithm for multivariate genomic prediction that is efficient in runtime and memory, applicable to unbalanced experimental designs, and exploits genetic correlations between environments to increase the accuracy of GEBV compared to univariate analyses. We proposed two algorithms, PEGS and THGS, that use randomized Gauss–Seidel to estimate marker effects and simultaneously estimate variance components, based on methods developed by [12, 13], respectively. Simulations were conducted to evaluate bias and accuracy of GEBV within environment and to compare them to those obtained by REML and a univariate approach. Bias and standard errors of estimates of heritabilities and genetic correlations were also evaluated to interpret the differences in bias and accuracy of GEBV between methods (Table 1).

PEGS and THGS were shown to be fast and memory-efficient algorithms for both balanced and unbalanced experimental designs, and had a much shorter runtime than REML using standard software implementations (Tables 3 and 4). Moreover, PEGS and THGS are scalable with the number of environments and markers. The reasons for the speed and efficiency of PEGS and THGS are that equations are solved by randomized Gauss–Seidel and that expectations of quadratic forms, shown in the denominator of Eqs. (5) and (6), are inexpensive to compute. These expectations do not require elements of the inverse of the left-hand side of the mixed-model equations as shown in [13]. Therefore, the system of equations essentially reduces to a $K\times K$ problem (Eq. 2) with complexity $O(K^3)$. When fitting hundreds to thousands of response variables, it is possible to linearize operations through full-conditional multivariate Gauss–Seidel algorithm presented in Appendix 5.

The number of iterations to convergence (Fig. 1) and runtime of PEGS and THGS decreased greatly by randomizing the marker order for updating marker effects (Table 4). This may be because randomization reduces dependencies of consecutively updated markers that stem from high linkage disequilibrium between adjacent markers on the same chromosome. With an increasing number of environments and markers, PEGS and THGS had reasonably short runtimes (Table 4, with randomization), which allows breeders to make decisions on time, and rerun genetic evaluations as data become available during harvest season.

For balanced designs, the number of iterations to convergence can be further reduced by modeling the eigenvectors of genotype scores, which completely removes dependencies among model effects. In addition, THGS becomes an exact method that yields unbiased estimates of genetic correlations and GEBV (section Exact THGS), and reduces the bias of estimates of heritabilities, as can be demonstrated for scenario 1 (Table 6). Matrix decomposition is also useful to analyze high-dimensional datasets with many factors ($P>>N$ problem), and to fit one or multiple kernels of different types within multivariate ridge regression models, for example, for modeling dominance, epistasis [37], and Gaussian or Arc-cosine relationships [21, 38]. The computing costs for matrix decomposition to obtain those eigenvectors, however, may outweigh the benefits for THGS as the number of individuals and markers in the analysis increases.

The trade-off for higher speed with PEGS and THGS is a slightly lower accuracy of GEBV of 0.01 compared to REML under realistic conditions when heritability was low and genetic correlations between environments were medium to high (Fig. 2a). PEGS and THGS exploited genetic correlations between environments under these conditions and had a higher accuracy of GEBV than the univariate approach (Fig. 2a, b). Only in the worst case, when all heritabilities and all genetic correlations between environments were low, did the benefit in accuracy of multivariate genomic prediction over the univariate approach vanish with PEGS and THGS (Fig. 2a, b). This occurred because PEGS and THGS resulted in notably higher standard errors of estimates of genetic correlations than REML (Fig. 7). Moreover, PEGS and THGS slightly underestimated heritabilities and slightly overestimated genetic correlations. The bias of GEBV, however, was close to zero and approached zero with an increasing number of lines per environment (Fig. 3, Table 5).

Residuals were treated as uncorrelated between environments for three reasons. First, the phenotypes come from different individuals that are assumed to have uncorrelated environmental effects. Second, epistatic effects, which are not captured by the marker effects in the model of Eq. (1), are assumed to have small covariances between environments. Third, the PEGS and THGS algorithms are faster because the absorption matrix ${\mathbf {M}}$, which is used in Eqs. (3) to (7), is block-diagonal with one block per environment, ${\mathbf {M}}_k$. And finally, fewer computations are required to update estimated marker effects when the residual covariance matrix is diagonal (see Eq. 2). If phenotypes come from multiple quantitative traits, residual covariances may need to be modeled to avoid further bias in the estimated genetic parameters and GEBV, which may increase runtime [13] and offset the computational advantage compared to REML. However, these covariances could be modeled with an additional random term that is constructed by the cross-product of sparse 0/1-incidence matrices for genotypes from different environments. Otherwise, the effect of neglecting the residual covariances on bias of estimates of genetic parameters and GEBV could be evaluated on a case-by-case basis.

Estimates of variances and covariances obtained by the methods PE and TH are unbiased when the mixed-model equations are weighted by the true variances and covariances as shown in Additional file 1, and Appendix 2. In practice, however, an iterative procedure starts with best guesses for genetic parameters and, thus, estimates are not expected to be unbiased, which is the same for REML or iterative MIVQUE [39]. As discussed in [12], estimates may be further biased when populations are under selection. In plant breeding, data are analyzed by breeding stage and thereby do not contain selection information, otherwise may be augmented with unselected genotypes [40, 41]. Yet, Ouweltjes et al. [42] and VanRaden and Jung [12] found that PE can be more suitable than TH to estimate variance components in populations under selection, but both methods were found to be slightly more biased than REML. These studies were performed using pedigree information and the bias was attributed to neglecting off-diagonals of the relationship matrix. To better understand this, the original quadratic form, $\hat{\varvec{\upbeta }}^{\prime}_k\hat{\varvec{\upbeta }}_k$, can be compared to $\tilde{\varvec{\upbeta }}^{\prime}_k\hat{\varvec{\upbeta }}_k$ from Eq. (5). For simplicity, only the univariate case and the method PE with $\tilde{\varvec{\upbeta }}_k = {\mathbf {Z}}^{\prime}_k{\mathbf {M}}_{k} {\mathbf {y}}_k$ is considered here. Using BLUP formulas [39], the quadratic forms can be written as:

9 $$\begin{aligned} \hat{\varvec{\upbeta }}^{\prime}_{k}\hat{\varvec{\upbeta }}_{k}&= \left( {\mathbf {y}}_{k}-{\mathbf {X}}\hat{{\mathbf {b}}}_{\text {GLS}_k}\right) ^{\prime}{\mathbf {V}}^{-1}_{k}{\mathbf {Z}}_{k}\sigma ^{2}_{\upbeta _k}\sigma ^{2}_{\upbeta _k}{\mathbf {Z}}^{\prime}_{k}{\mathbf {V}}^{-1}_{k}\left( {\mathbf {y}}_{k}-{\mathbf {X}}\hat{{\mathbf {b}}}_{\text {GLS}_{k}}\right) , \end{aligned}$$

and

10 $$\begin{aligned} \tilde{\varvec{\upbeta }}^{\prime}_{k}\hat{\varvec{\upbeta }}_{k}&= {\mathbf {y}}^{\prime}_{k}{\mathbf {M}}_{k}{\mathbf {Z}}_{k} \hat{\varvec{\upbeta }}_{k} \nonumber \\&=\left( {\mathbf {y}}_{k}-{\mathbf {X}}\hat{{\mathbf {b}}}_{\text {LS}_{k}}\right) ^{\prime}{\mathbf {Z}}_{k}\sigma ^{2}_{\upbeta _k}{\mathbf {Z}}^{\prime}_{k}{\mathbf {V}}^{-1}_{k}\left( {\mathbf {y}}_{k}-{\mathbf {X}}\hat{{\mathbf {b}}}_{\text {GLS}_{k}}\right) , \end{aligned}$$

where ${\mathbf {V}}^{-1}_k$ is the inverse of the variance-covariance matrix of ${\mathbf {y}}_k$, ${\mathbf {V}}_k = {\mathbf {Z}}_k{\mathbf {Z}}^{\prime}_k\sigma ^2_{\upbeta _k}+{\mathbf {I}}\sigma ^2_{e_k}$, and $\hat{{\mathbf {b}}}_{\text {GLS}_k}$ and $\hat{{\mathbf {b}}}_{\text {LS}_k}$ are the generalized least squares and least squares estimators, respectively, of ${\mathbf {b}}$. Thus in $\tilde{\varvec{\upbeta }}_k$, the matrix ${\mathbf {V}}^{-1}_k$, which contains genomic relationships between individuals, i.e., ${\mathbf {Z}}_k{\mathbf {Z}}^{\prime}_k$, is not used to weigh ${\mathbf {y}}_k$, or to estimate fixed effects ($\hat{{\mathbf {b}}}_{\text {LS}_k}$) or random effects. However, THGS in combination with principal components or eigenvector regression provides the exact estimates of variance and covariance components for populations under selection.

PEGS and THGS should be evaluated against alternative methods for modeling phenotypes from multiple environments. These are compound symmetry and extended factor analytic (XFA) models [43]. Compound symmetry models fit a term for the average genetic effect of an individual across environments and another term for the specific environmental effects for an individual. As each term is modeled with only one variance, this model assumes that the genetic correlations between all pairs of environments are identical. The difference between that single correlation and the true correlation between any pair of environments can be regarded as bias. The XFA model fits more parameters than the compound symmetry model to reduce this bias, but less parameters than an unstructured multivariate model that fits a correlation for each pair of environments, and thus balances bias and precision of estimated genetic correlations. Therefore, these two alternative models tend to bias estimates of genetic correlations between environments and are expected to decrease accuracy of GEBV compared to estimating genetic correlations between all pairs of environments, unless the amount of genetic information is limited.

The iterative algorithm of PEGS and THGS differs from that of REML and Bayesian Gibbs sampling. In each iteration of REML, the mixed-model equations are fully solved to obtain estimates of the model effects conditional on the current variance components of that iteration. The estimated model effects are then used to update the variance components and a new iteration begins, unless the change in variance components is small. In PEGS and THGS, in contrast, the model effects are merely updated, not solved, before variance components are updated and a new iteration begins. In Bayesian Gibbs sampling, similar computations are conducted in each iteration as in PEGS and THGS. However, rather than converging directly to a solution within a small number of iterations, the Gibbs algorithm samples from the posterior for thousands of iterations and, therefore, must have longer runtimes.

## Conclusions

PEGS and THGS are fast, memory-efficient, and reliable algorithms for genomic prediction for both balanced and unbalanced experimental designs. They are scalable with an increasing number of response variables and markers. Their runtime is much shorter than for REML and Gibbs sampling. For balanced designs, THGS provides unbiased GEBV and estimates of genetic correlations if only an intercept is modeled, and eigenvalue decomposition is feasible. Without eigenvalue decomposition, the accuracy of GEBV obtained using PEGS and THGS is slightly lower than of GEBV obtained using REML, but higher than that of univariate THGS under realistic genetic correlations between environments. Estimates of genetic parameters obtained using PEGS and THGS have little bias, but their standard errors are larger than for REML. More studies are needed to evaluate the PEGS and THGS algorithms for unbalanced datasets with selection.

### Supplementary Information

**Additional file 1.** An R implementation of PEGS.

**Additional file 2.** Deterministic calculations to study estimators of variance components using *Tilde-Hat* or *Pseudo-Expectation*.

**Additional file 3.** Scenario 1 with more environments.

**Additional file 4.** Estimated covariances for different degrees of balanced data.

**Additional file 5.** Summary of scenario 2 and balanced case.

**Additional file 6.** R code demonstrating unbiasedness of PEGS.

## Data Availability

Genotypic data of the wheat dataset are available in the R package BGLR using the command data(wheat,package=“BGLR”), and genotypic data of the SoyNAM dataset are available in the R package SoyNAM using the command data ← SoyNAM::ENV(). An implementation of PEGS is provided in the R package bWGR (version 2.0), function mrr. A demonstration of PEGS unbiasedness is provided in the Additional file 5, and an R implementation is provided in Additional file 6.

## Appendix 1: Efficient calculation of ${\mathbf {Z}}^{\prime}_{k} {\mathbf {M}}_{k} {\mathbf {Z}}_{k}$ and ${\mathbf {M}}_{k}{\mathbf {y}}_k$

Only the diagonal elements of ${\mathbf {Z}}^{\prime}_{k} {\mathbf {M}}_{k} {\mathbf {Z}}_{k}$ are needed as matrix ${\mathbf {D}}_k$ is diagonal (Eq. 4). They can be computed one at a time for environment *k* and marker *j* as:

$${\mathbf {z}}^{\prime}_{jk} {\mathbf {M}}_{jk} {\mathbf {z}}_{k} = {\mathbf {z}}_{jk}^{\prime}{\mathbf {z}}_{jk} - {\mathbf {z}}_{jk}^{\prime}{\mathbf {X}}_{k}\left( {\mathbf {X}}^{\prime}_{k} {\mathbf {X}}_{k}\right) ^{-1} {\mathbf {X}}^{\prime}_{k} {\mathbf {z}}_{jk}$$

where $({\mathbf {X}}^{\prime}_{k} {\mathbf {X}}_{k})^{-1}$ is computed once before iterations start. Likewise, ${\mathbf {M}}_{k}{\mathbf {y}}_k$ of Eq. (7) can be obtained once as:

$${\mathbf {M}}_{k}{\mathbf {y}}_k = {\mathbf {y}}_k - {\mathbf {X}}_{k}\left( {\mathbf {X}}^{\prime}_{k} {\mathbf {X}}_{k}\right) ^{-1} {\mathbf {X}}^{\prime}_{k} {\mathbf {y}}_k = {\mathbf {y}}_k - {\mathbf {X}}_{k} {\hat{\mathbf{b}}}_{LS_k},$$

where $\hat{\mathbf {b}}_{LS_k}$ denotes the Least Squares estimate of ${\mathbf {b}}$.

## Appendix 2: Expected value of $\tilde{\varvec{\upbeta }}^{\prime}_k\hat{\varvec{\upbeta }}_k$

Let $\tilde{\varvec{\upbeta }}_k = {\mathbf {D}}^{-1}_k{\mathbf {Z}}^{\prime}_k{\mathbf {M}}_k{\mathbf {y}}_k$ and ${\mathbf {M}}_k = {\mathbf {I}}_k - {\mathbf {X}}_k({\mathbf {X}}^{\prime}_k{\mathbf {X}}_k)^{-1}{\mathbf {X}}_k$, as defined in the section *Solving variances and covariances*, and let $\hat{\varvec{\upbeta }}_k = \sigma ^2_{\upbeta _k}{\mathbf {Z}}^{\prime}_k{\mathbf {P}}_k{\mathbf {y}}_k$ be the best linear unbiased predictor (BLUP) of $\varvec{\upbeta }$ [39], where ${\mathbf {P}}_k = {\mathbf {V}}^{-1}_k[{\mathbf {I}}_k-{\mathbf {X}}_k({\mathbf {X}}^{\prime}_k{\mathbf {V}}^{-1}_k{\mathbf {X}}_k)^{-1}{\mathbf {X}}_k{\mathbf {V}}^{-1}_k]$ and $E(\hat{\varvec{\upbeta }}) = {\mathbf {0}}$. Then, the expected value of the bilinear form $\tilde{\varvec{\upbeta }}^{\prime}_k\hat{\varvec{\upbeta }}_k$ [44] is:

$$\begin{aligned} E(\tilde{\varvec{\upbeta }}^{\prime}_{k}\hat{\varvec{\upbeta }}_{k})&= tr(Cov(\tilde{\varvec{\upbeta }}_{k}, \hat{\varvec{\upbeta }}^{\prime}_{k})) + E(\tilde{\varvec{\upbeta }}_{k})^{\prime}E(\hat{\varvec{\upbeta }}_{k})\\&= tr\left( {\mathbf {D}}^{-1}_{k}{\mathbf {Z}}^{\prime}_{k}{\mathbf {M}}_{k}{\mathbf {V}}_{k}{\mathbf {P}}_{k}{\mathbf {Z}}_{k}\sigma ^{2}_{\upbeta _k}\right) \\&= tr\left( {\mathbf {D}}^{-1}_{k}{\mathbf {Z}}^{\prime}_{k}{\mathbf {M}}_{k}{\mathbf {Z}}_{k}\right) \sigma ^{2}_{\upbeta _k}, \end{aligned}$$

because $${\mathbf {M}}_k{\mathbf {V}}_k{\mathbf {P}}_k = {\mathbf {M}}_k$$. Hence,

$$\begin{aligned} {\hat{\sigma }}^{2}_{\upbeta _k}&= \frac{\tilde{\varvec{\upbeta }}^{\prime}_{k}\hat{\varvec{\upbeta }}_{k}}{tr\left( {\mathbf {D}}^{-1}_{k}{\mathbf {Z}}^{\prime}_{k}{\mathbf {M}}_{k}{\mathbf {Z}}_{k}\right) }, \end{aligned}$$

and $E({\hat{\sigma }}^2_{\upbeta _k}) = \sigma ^2_{\upbeta _k}$. The extension to using $\hat{\varvec{\upbeta }}_k$ from a multivariate BLUP is presented in Additional file 1.

## Appendix 3: Equivalence of $\hat{\varvec{\upbeta }}$ and $\tilde{\varvec{\upbeta }}$ using EVD

Let the eigenvalue decomposition of ${\mathbf {Z}}^{\prime}_k{\mathbf {Z}}_k$ be ${\mathbf {U}}_k\varvec{\Lambda }_k{\mathbf {U}}^{\prime}_k$, where ${\mathbf {U}}_k$ is an orthonormal matrix of eigenvectors with the property ${\mathbf {U}}^{\prime}_k{\mathbf {U}}_k = {\mathbf {U}}_k{\mathbf {U}}^{\prime}_k = {\mathbf {I}}_m$, and $\varvec{\Lambda }_k$ is a diagonal matrix of eigenvalues. The principal component regression [18] can be written as:

$$\begin{aligned} {\mathbf {y}}_{k}&= {\mathbf {1}}\mu _{k} + {\mathbf {Z}}_{k}{\mathbf {U}}_{k}{\mathbf {U}}^{\prime}_{k}\varvec{\upbeta }_{k} + {\mathbf {e}}_{k}\\&= {\mathbf {1}}\mu _{k} +\check{{\mathbf {Z}}}_{k}\check{\varvec{\upbeta }}_{k} + {\mathbf {e}}_{k}, \end{aligned}$$

where $\tilde{{\mathbf {Z}}}_{k} = {\mathbf {Z}}_{k}{\mathbf {U}}_{k}$ and $\check{\varvec{\upbeta }}_{k} = {\mathbf {U}}^{\prime}_{k}\varvec{\upbeta }_{k}$. Let the estimate of $\check{\varvec{\upbeta }}_{k}$ be $\tilde{\varvec{\upbeta }}_{k} = {\mathbf {D}}^{-1}_{k}\check{{\mathbf {Z}}}^{\prime}_{k}{\mathbf {y}}_{k}$ similar to Eq. (8), where ${\mathbf {M}}_{k}$ was omitted because ${\mathbf {Z}}_k$ and ${\mathbf {y}}_k$ are assumed centered. Then, defining $\lambda _{k} = \sigma ^{2}_{e_{k}}/\sigma ^{2}_{\upbeta _{k}}$, and using $({\mathbf {U}}_k)^{-1} = {\mathbf {U}}^{\prime}_k$ and $({\mathbf {U}}^{\prime}_k)^{-1} = {\mathbf {U}}_k$,

$$\begin{aligned} \hat{\varvec{\upbeta }}_k&= \left( {\mathbf {Z}}^{\prime}_{k}{\mathbf {Z}}_{k} + {\mathbf {I}}_{m}\lambda _{k}\right) ^{-1}{\mathbf {Z}}^{\prime}_{k}{\mathbf {y}}_{k}\\&= {\mathbf {U}}_{k}\tilde{\varvec{\upbeta }}_{k}\\&= {\mathbf {U}}_{k}{\mathbf {D}}^{-1}_{k}\check{{\mathbf {Z}}}^{\prime}_{k}{\mathbf {y}}_{k}\\&= {\mathbf {U}}_{k}\left( \varvec{\Lambda }_{k}+{\mathbf {I}}_{m}\lambda _{k}\right) ^{-1}\check{{\mathbf {Z}}}_k{\mathbf {y}}_{k}\\&= {\mathbf {U}}_{k}\left[ {\mathbf {U}}^{\prime}_{k}{\mathbf {U}}_{k}\left( \varvec{\Lambda }_{k}+{\mathbf {I}}_{m}\lambda _{k}\right) {\mathbf {U}}^{\prime}_{k}{\mathbf {U}}_{k}\right] ^{-1}\check{{\mathbf {Z}}}^{\prime}_k{\mathbf {y}}_k\\&= {\mathbf {U}}_{k}\left[ {\mathbf {U}}^{\prime}_{k}\left( {\mathbf {U}}_{k}\varvec{\Lambda }_k{\mathbf {U}}^{\prime}_k+{\mathbf {I}}_m\lambda _{k}\right) {\mathbf {U}}_{k}\right] ^{-1}\check{{\mathbf {Z}}}^{\prime}_{k}{\mathbf {y}}_{k}\\&= {\mathbf {U}}_{k}{\mathbf {U}}^{\prime}_{k}\left( {\mathbf {Z}}^{\prime}_{k}{\mathbf {Z}}_{k}+{\mathbf {I}}_{m}\lambda _k\right) ^{-1}{\mathbf {U}}_{k}\tilde{{\mathbf {Z}}}^{\prime}_{k}{\mathbf {y}}_{k}\\&= \left( {\mathbf {Z}}^{\prime}_{k}{\mathbf {Z}}_{k}+{\mathbf {I}}_{m}\lambda _k\right) ^{-1}{\mathbf {U}}_{k}{\mathbf {U}}^{\prime}_{k}{\mathbf {Z}}^{\prime}_{k}{\mathbf {y}}_{k}\\&= ({\mathbf {Z}}^{\prime}_{k}{\mathbf {Z}}_{k}+{\mathbf {I}}_{m}\lambda _{k})^{-1}{\mathbf {Z}}^{\prime}_{k}{\mathbf {y}}_{k}. \end{aligned}$$

## Appendix 4: Polygenic model using EVD

The model can be written as:

11 $${\mathbf {y}} = {\mathbf {X}}{\mathbf {b}} + {\mathbf {g}} + {\mathbf {e}},$$

where ${\mathbf {y}}$, ${\mathbf {X}}$, ${\mathbf {b}}$, and ${\mathbf {e}}$ are defined as in the section *statistical model*, and ${\mathbf {g}}$ is a vector of breeding values that can be partitioned into ${\mathbf {g}}^{\prime} = [{\mathbf {g}}^{\prime}_1 ~{\mathbf {g}}^{\prime}_2 ~\ldots ~{\mathbf {g}}^{\prime}_K$]. It is assumed to be multivariate normal-distributed with mean zero and variance $\varvec{\Sigma }_g\otimes {\mathbf {G}}$, where $\varvec{\Sigma }_g$ is a *K* x *K* variance-covariance matrix of breeding values for *K* environments and ${\mathbf {G}}$ is the genomic relationship matrix. The eigenvalue decomposition of this matrix can be written as ${\mathbf {G}} = {\mathbf {U}}\varvec{\Lambda }{\mathbf {U}}^{\prime}$, where ${\mathbf {U}}$ contains orthogonal eigenvectors and $\varvec{\Lambda }$ is a diagonal matrix that contains eigenvalues. To diagonalize ${\mathbf {G}}$, model (11) was tranformed by ${\mathbf {T}} = {\mathbf {1}}_K\otimes {\mathbf {U}}^{\prime}$, where ${\mathbf {1}}_K$ is a *K* vector of 1s, hence:

$$\begin{aligned} {\mathbf {T}}{\mathbf {y}}&= {\mathbf {T}}{\mathbf {X}}{\mathbf {b}} + {\mathbf {T}}{\mathbf {g}} + {\mathbf {T}}{\mathbf {e}}\\&= \tilde{{\mathbf {X}}}{\mathbf {b}} + \tilde{{\mathbf {g}}} + \tilde{{\mathbf {e}}}, \end{aligned}$$

where $$\tilde{{\mathbf {g}}}\sim N({\mathbf {0}}, \varvec{\Sigma }_g\otimes \varvec{\Lambda })$$ and $$\tilde{{\mathbf {e}}}\sim N({\mathbf {0}}, \oplus ^{K}_{i=1}{\mathbf {I}}\sigma ^{2}_{e_k})$$.

## Appendix 5: Full-conditional Gauss–Seidel solution

Equation (2) can be rearranged to reduce the multivariate Gauss–Seidel solver into a univariate algorithm, as an extension of the algorithm in [15]. This circumvents the inverse in Eq. (2), but may have slower convergence. The estimated effect of marker *j* and environment *k* is updated as:

$${\hat{\upbeta }}^{(t+1)}_{jk}|\hat{\varvec{\upbeta }}^{(t)}_j,\hat{\varvec{\Sigma }}_\upbeta = \frac{ {\mathbf {z}}_{jk}^{\prime}\hat{{\mathbf {e}}}_k + {\mathbf {z}}^{\prime}_{jk} {\mathbf {z}}_{jk} {\hat{\upbeta }}_{jk}^{(t)} - {\hat{\sigma }}^{2}_{e_k}\sum ^K_{l=1, l\ne k}\hat{\varvec{\Sigma }}^{-1}_{\upbeta _{kl}}\cdot {\hat{\upbeta }}^{(t)}_{jl}}{ {\mathbf {z}}_{jk} ^{\prime} {\mathbf {z}}_{jk} + {\hat{\sigma }}^2_{e_k}{\hat{\sigma }}_\upbeta ^{kk} }$$

where ${\hat{\sigma }}_\upbeta ^{kk}$ is the *kk* element of $\hat{\varvec{\Sigma }}^{-1}_\upbeta$. The update of ${\hat{\upbeta }}^{(t+1)}_{jk}$ is followed by the update of residuals of environment *k* as:

$$\hat{{\mathbf {e}}}^{(new)}_{k} = \hat{{\mathbf {e}}}^{(old)}_{k}-{\mathbf {z}}_{jk}({\hat{\upbeta }}_{jk}^{(t+1)}-{\hat{\upbeta }}_{jk}^{(t)}).$$
