# Supplementary material for: A new approach fits multivariate genomic prediction models efficiently
Source: Genet Sel Evol. 2022 Jun 17;54:45. doi: 10.1186/s12711-022-00730-w (PMC9204867; doi:10.1186/s12711-022-00730-w)
Supplement: Supplementary file 1 — Additional file 1. An R implementation of PEGS. [file 12711_2022_730_MOESM1_ESM.pdf]

An R implementation of PEGS

Alencar Xavier and David Habier

May 6, 2022

The code below can be pasted and loaded in using the Rcpp package in R. It is a function that takes two inputs: Y and X. The input Y is numeric matrix of phenoypes where columns corresponds to traits and rows are individuals, where missing values are allowed. The input X is numeric matrix of genomic information where columns corresponds to markers and rows are individuals, where missing values are not allowed.

```
// [[Rcpp::depends(RcppEigen)]]
#include <RcppEigen.h>
#include <iostream>
#include <random>

// [[Rcpp::export]]
SEXP PEGS(Eigen::MatrixXd Y, Eigen::MatrixXd X){
 int J, maxit = 1000, k = Y.cols(), n0 = Y.rows(), p = X.cols();
 // Incidence matrix Z
 Eigen::MatrixXd Z(n0,k);
 for(int i=0; i<n0; i++){ for(int j=0; j<k; j++){
  if(std::isnan(Y(i,j))){Z(i,j) = 0.0; Y(i,j) = 0.0;}else{ Z(i,j) = 1.0;}}}
 // Count observations per trait
 Eigen::VectorXd n = Z.colwise().sum();
 Eigen::VectorXd iN = n.array().inverse();
 // Centralize y
 Eigen::VectorXd mu=Y.colwise().sum(); mu=mu.array()*iN.array(); Eigen::MatrixXd y(n0,k);
 for(int i=0; i<k; i++){ y.col(i) = (Y.col(i).array()-mu(i)).array()*Z.col(i).array();}
 // Sum of squares of X abd Tr(XSX)
 Eigen::MatrixXd XX(p,k), XSX(p,k);
 for(int i=0; i<p; i++){ XX.row(i) = X.col(i).array().square().matrix().transpose()*Z;}
 for(int i=0; i<p; i++){ XSX.row(i) = XX.row(i).transpose().array()*iN.array() -
  ((X.col(i).transpose()*Z).transpose().array()*iN.array()).square();}
 Eigen::VectorXd MSx = XSX.colwise().sum();
 Eigen::VectorXd TrXSX = n.array()*MSx.array();
 // Variances
 iN = (n.array()-1).inverse();
 Eigen::VectorXd h2(k), vy = y.colwise().squaredNorm(); vy = vy.array()*iN.array();
 Eigen::VectorXd ve = vy*0.5; Eigen::VectorXd iVe = ve.array().inverse();
 Eigen::MatrixXd vb(k,k), iG(k,k), TildeHat(k,k), GC(k,k);
 vb = (ve.array()/MSx.array()).matrix().asDiagonal(); iG = vb.inverse();
```

```
// Initialize coefficient matrices;
Eigen::MatrixXd tilde = X.transpose()*y;
Eigen::MatrixXd LHS(k,k), b=Eigen::MatrixXd::Zero(p,k), e(n0,k); e=y*1.0;
Eigen::VectorXd RHS(k), b0(k), b1(k);
// RGS
std::vector<int> RGSvec(p);  for(int j=0; j<p; j++){RGSvec[j]=j;}
std::random_device rd;  std::mt19937 g(rd());
// Convergence control
double deflate = 1.0, deflateMax = 0.75, cnv = 10.0, logtol = -10.0;
Eigen::MatrixXd beta0(p,k), A(k,k); int numit = 0;
// Loop
while(numit<maxit){
 beta0 = b*1.0; // Store current values
 std::shuffle(RGSvec.begin(), RGSvec.end(), g); // Randomization
 for(int j=0; j<p; j++){ // Gauss-Seidel loop
  J = RGSvec[j]; b0 = b.row(J)*1.0;
  LHS = iG;  LHS.diagonal() += (XX.row(J).transpose().array() * iVe.array()).matrix();
  RHS = (X.col(J).transpose()*e).array() + XX.row(J).array()*b0.transpose().array();
  RHS = RHS.array() *iVe.array();
  b1 = LHS.llt().solve(RHS); b.row(J) = b1; // Update coefficient
  // Update residuals
  e = (e-(X.col(J)*(b1-b0).transpose()).cwiseProduct(Z)).matrix();}
 // Estimate residual variances
 ve = (e.cwiseProduct(y)).colwise().sum();
 ve = ve.array()*iN.array(); iVe = ve.array().inverse();
 // Genetic variance
 TildeHat = b.transpose()*tilde;
 for(int i=0; i<k; i++){for(int j=0; j<k; j++){
  if(i==j){ vb(i,i) = TildeHat(i,i)/TrXSX(i); }else{
  vb(i,j) = (TildeHat(i,j)+TildeHat(j,i))/(TrXSX(i)+TrXSX(j));}}}
 // Bending
 A = vb*1.0;
 for(int i=0; i<k; i++){for(int j=0; j<k; j++){ if(i!=j){A(i,j) *= deflate;}}}
 while(A.llt().info()==Eigen::NumericalIssue && deflate>deflateMax){
   Rcpp::Rcout << "Bend at "<< numit << "\n";  deflate = deflate - 0.01;
   for(int i=0;i<k;i++){for(int j=0; j<k; j++){if(i!=j){A(i,j) = vb(i,j)*deflate;}}}}
 iG = A.inverse();
 // Print status
 cnv = log10((beta0.array()-b.array()).square().sum());  ++numit;
 if(numit%100==0){Rcpp::Rcout<<"Iter: "<< numit << " |Conv: "<< cnv << "\n";}
 if(cnv<logtol){break;}}
// Fitting the model, genetic correlations
h2 = 1 - ve.array()/vy.array(); Eigen::MatrixXd hat = X * b;
for(int i=0; i<k; i++){hat.col(i) = hat.col(i).array() + mu(i);}
for(int i=0;i<k;i++){for(int j=0;j<k;j++){GC(i,j)=vb(i,j)/(sqrt(vb(i,i)*vb(j,j)));}}
// Output
return Rcpp::List::create(Rcpp::Named("mu")=mu, Rcpp::Named("b")=b,
                          Rcpp::Named("hat")=hat, Rcpp::Named("h2")=h2,
                          Rcpp::Named("GC")=GC, Rcpp::Named("bend")=deflate,
                          Rcpp::Named("cnv")=cnv);}
```
