# Supplementary material for: A new approach fits multivariate genomic prediction models efficiently
Source: Genet Sel Evol. 2022 Jun 17;54:45. doi: 10.1186/s12711-022-00730-w (PMC9204867; doi:10.1186/s12711-022-00730-w)
Supplement: Supplementary file 2 — Additional file 2. Deterministic calculations to study estimators of variance components using Tilde-Hat or Pseudo-Expectation. [file 12711_2022_730_MOESM2_ESM.pdf]

Deterministic calculations to study estimators of variance components using *Tilde-Hat* or *Pseudo-Expectation*

Alencar Xavier and David Habier

May 12, 2022

A deterministic tool was coded in R to demonstrate that variances and covariances estimated by equations (5) and (6) of the main manuscript are unbiased for different heritabilities and genetic correlations. More specifically, the objective is to derive expected values of the estimators for variances and covariances and compare them to the true, given parameters.

The calculations are based on the bivariate model

$$\begin{bmatrix} \boldsymbol{y}_k \\ \boldsymbol{y}_{k'} \end{bmatrix} = \begin{bmatrix} \mathbf{X}_k & 0 \\ 0 & \mathbf{X}_{k'} \end{bmatrix} \begin{bmatrix} \boldsymbol{b}_k \\ \boldsymbol{b}_{k'} \end{bmatrix} + \begin{bmatrix} \mathbf{Z}_k & 0 \\ 0 & \mathbf{Z}_{k'} \end{bmatrix} \begin{bmatrix} \boldsymbol{\beta}_k \\ \boldsymbol{\beta}_{k'} \end{bmatrix} + \begin{bmatrix} \boldsymbol{e}_k \\ \boldsymbol{e}_{k'} \end{bmatrix}$$
$$\boldsymbol{y} = \mathbf{X}\boldsymbol{b} + \mathbf{Z}\boldsymbol{\beta} + \boldsymbol{e},$$

where $\boldsymbol{y}_k$ and $\boldsymbol{y}_{k'}$ are vectors of phenotypes, $\mathbf{X}_k$ and $\mathbf{X}_{k'}$ are incidence matrices for fixed effects, $\mathbf{Z}_k$ and $\mathbf{Z}_{k'}$ are matrices of centered marker scores, $\boldsymbol{\beta}_k$ and $\boldsymbol{\beta}_{k'}$ are vectors of random marker effects, and $\boldsymbol{e}_k$ and $\boldsymbol{e}_{k'}$ are vectors of random residual effects, all for the two environments k and k', respectively. The expected value of $\boldsymbol{y}$ is $\mathbf{X}\boldsymbol{b}$, the variances of $\boldsymbol{\beta}$ and $\boldsymbol{e}$ are $Var(\boldsymbol{\beta}) = \mathbf{G} = \boldsymbol{\Sigma}_\beta \otimes \mathbf{I}_m$ and $Var(\boldsymbol{e}) = \mathbf{R} = \mathbf{I}_k\sigma^2_k \oplus \mathbf{I}_{k'}\sigma^2_{k'}$, where $\boldsymbol{\Sigma}_\beta$ is the variance-covariance matrix of marker effects, $\mathbf{I}_m$, $\mathbf{I}_k$ and $\mathbf{I}_{k'}$ are identity matrices, $\sigma^2_{e_k}$ and $\sigma^2_{e_{k'}}$ are residual variances for environments k and k', and $\oplus$ is the direct matrix sum. The variance of $\boldsymbol{y}$ is $\mathbf{V} = \mathbf{Z}\mathbf{G}\mathbf{Z}' + \mathbf{R}$. The elements of $\boldsymbol{\Sigma}_\beta$ are calculated as

$$\boldsymbol{\Sigma}_\beta = \begin{bmatrix} \sigma^2_{\beta_k} & \sigma_{\beta_{kk'}} \\ \sigma_{\beta_{kk'}} & \sigma^2_{\beta_{k'}} \end{bmatrix}$$
$$= \frac{1}{m} \begin{bmatrix} \sigma^2_{g_k} & \sigma_{g_{kk'}} \\ \sigma_{g_{kk'}} & \sigma^2_{g_{k'}} \end{bmatrix},$$

where m is the number of markers, $\sigma^2_{g_k}$ and $\sigma^2_{g_{k'}}$ are the additive-genetic variances of environments k and k', and $\sigma_{g_{kk'}}$ is the additive-genetic covariance between the two environments. These additive-genetic parameters are provided as input to the deterministic calculations and they are compared to the expected

values of their estimators to show that these are unbiased.

Marker effects are estimated by Best Linear Unbiased Prediction [39] as

$$\hat{\boldsymbol{\beta}} = Cov(\boldsymbol{\beta}, \boldsymbol{y}')\mathbf{P}\boldsymbol{y},$$

where $\mathbf{P} = \mathbf{V}^{-1}[\mathbf{I} - \mathbf{X}(\mathbf{X}'\mathbf{V}^{-1}\mathbf{X})^{-1}\mathbf{X}'\mathbf{V}^{-1}]$ and

$$\begin{aligned} Cov(\boldsymbol{\beta}, \boldsymbol{y}') &= \begin{bmatrix} \sigma^2_{\beta_k}\mathbf{Z}'_k & \sigma_{\beta_{kk'}}\mathbf{Z}'_{k'} \\ \sigma_{\beta_{kk'}}\mathbf{Z}'_k & \sigma^2_{\beta_{k'}}\mathbf{Z}'_{k'} \end{bmatrix} \\ &= \begin{bmatrix} \mathbf{V}_{\beta_k y} \\ \mathbf{V}_{\beta_{k'} y} \end{bmatrix}. \end{aligned}$$

The expected value of $\hat{\boldsymbol{\beta}} = \mathbf{0}$ as $\mathbf{PX} = \mathbf{0}$. For estimating variances and covariances of marker effects $\tilde{\boldsymbol{\beta}}_k = \mathbf{D}_k^{-1}\mathbf{Z}'_k\mathbf{M}_k\boldsymbol{y}_k$ and $\tilde{\boldsymbol{\beta}}_{k'} = \mathbf{D}_{k'}^{-1}\mathbf{Z}'_{k'}\mathbf{M}_{k'}\boldsymbol{y}_{k'}$ are used in

$$\hat{\sigma}^2_{\beta_k} = \frac{\tilde{\boldsymbol{\beta}}'_k\hat{\boldsymbol{\beta}}_k}{tr(\mathbf{D}_k^{-1}\mathbf{Z}'_k\mathbf{M}_k\mathbf{Z}_k)},$$

and

$$\hat{\sigma}_{\beta_{kk'}} = \frac{\tilde{\boldsymbol{\beta}}'_k\hat{\boldsymbol{\beta}}_{k'} + \tilde{\boldsymbol{\beta}}'_{k'}\hat{\boldsymbol{\beta}}_k}{tr(\mathbf{D}_k^{-1}\mathbf{Z}'_k\mathbf{M}_k\mathbf{Z}_k) + tr(\mathbf{D}_{k'}^{-1}\mathbf{Z}'_{k'}\mathbf{M}_{k'}\mathbf{Z}_{k'})},$$

respectively. The expected value of $\tilde{\boldsymbol{\beta}}'_k\hat{\boldsymbol{\beta}}_k$ [44] is

$$\begin{aligned} E(\tilde{\boldsymbol{\beta}}'_k\hat{\boldsymbol{\beta}}_k) &= tr(Cov(\tilde{\boldsymbol{\beta}}_k, \hat{\boldsymbol{\beta}}'_k)) + E(\tilde{\boldsymbol{\beta}}_k)'E(\hat{\boldsymbol{\beta}}_k) \\ &= tr(\mathbf{D}_k^{-1}\mathbf{Z}'_k\mathbf{M}_k Cov(\boldsymbol{y}_k, \boldsymbol{y}')\mathbf{P}\mathbf{V}'_{\beta_k y}) \end{aligned}$$

and the expected values of $\tilde{\boldsymbol{\beta}}'_k\hat{\boldsymbol{\beta}}_{k'}$ and $\tilde{\boldsymbol{\beta}}'_{k'}\hat{\boldsymbol{\beta}}_k$ are

$$E(\tilde{\boldsymbol{\beta}}'_k\hat{\boldsymbol{\beta}}_{k'}) = tr(\mathbf{D}_k^{-1}\mathbf{Z}'_k\mathbf{M}_k Cov(\boldsymbol{y}_k, \boldsymbol{y}')\mathbf{P}\mathbf{V}'_{\beta_{k'} y})$$

and

$$E(\tilde{\boldsymbol{\beta}}'_{k'}\hat{\boldsymbol{\beta}}_k) = tr(\mathbf{D}_{k'}^{-1}\mathbf{Z}'_{k'}\mathbf{M}_{k'} Cov(\boldsymbol{y}_{k'}, \boldsymbol{y}')\mathbf{P}\mathbf{V}'_{\beta_k y}),$$

respectively. The matrices $Cov(\boldsymbol{y}_k, \boldsymbol{y}')$ and $Cov(\boldsymbol{y}_{k'}, \boldsymbol{y}')$ are row-partitions of $\mathbf{V}$. The expected values of $\hat{\sigma}^2_{\beta_k}$ and $\hat{\sigma}_{\beta_{kk'}}$ are

$$E(\hat{\sigma}^2_{\beta_k}) = \frac{tr(\mathbf{D}_k^{-1}\mathbf{Z}'_k\mathbf{M}_k Cov(\boldsymbol{y}_k, \boldsymbol{y}')\mathbf{P}\mathbf{V}'_{\beta_k y})}{tr(\mathbf{D}_k^{-1}\mathbf{Z}'_k\mathbf{M}_k\mathbf{Z}_k)}, \quad (1)$$

and

$$E(\hat{\sigma}_{\beta_{kk'}}) = \frac{tr(\mathbf{D}_k^{-1}\mathbf{Z}'_k\mathbf{M}_k Cov(\boldsymbol{y}_k, \boldsymbol{y}')\mathbf{P}\mathbf{V}'_{\beta_{k'} y}) + tr(\mathbf{D}_{k'}^{-1}\mathbf{Z}'_{k'}\mathbf{M}_{k'} Cov(\boldsymbol{y}_{k'}, \boldsymbol{y}')\mathbf{P}\mathbf{V}'_{\beta_k y})}{tr(\mathbf{D}_k^{-1}\mathbf{Z}'_k\mathbf{M}_k\mathbf{Z}_k) + tr(\mathbf{D}_{k'}^{-1}\mathbf{Z}'_{k'}\mathbf{M}_{k'}\mathbf{Z}_{k'})}, \quad (2)$$

respectively. The expected additive-genetic variance and covariance are

$$
\begin{aligned}
E(\hat{\sigma}^2_{g_k}) &= m \cdot E(\hat{\sigma}^2_{\beta_k}) \\
E(\hat{\sigma}^2_{g_{k'}}) &= m \cdot E(\hat{\sigma}^2_{\beta_{k'}}) \\
E(\hat{\sigma}_{g_{kk'}}) &= m \cdot E(\hat{\sigma}_{\beta_{kk'}}).
\end{aligned}
$$

and biases are

$$
\begin{aligned}
\text{Bias}(\hat{\sigma}^2_{g_k}) &= E(\hat{\sigma}^2_{g_k}) - \sigma^2_{g_k} \\
\text{Bias}(\hat{\sigma}^2_{g_{k'}}) &= E(\hat{\sigma}^2_{g_{k'}}) - \sigma^2_{g_{k'}} \\
\text{Bias}(\hat{\sigma}_{g_{kk'}}) &= E(\hat{\sigma}_{g_{kk'}}) - \sigma_{g_{kk'}}.
\end{aligned}
$$

In the R code, shown in section 5, $\mathbf{D} = \mathbf{I}_m$. Biases are zero for different additive-genetic variances and covariances as well as residual variances. Thus, by inspection, $\mathbf{M}_k Cov(\boldsymbol{y}_k, \boldsymbol{y}')\mathbf{P} = [\mathbf{M}_k \vdots \mathbf{0}]$ and $\mathbf{M}_{k'} Cov(\boldsymbol{y}_{k'}, \boldsymbol{y}')\mathbf{P} = [\mathbf{0} \vdots \mathbf{M}_{k'}]$, so that equations (1) and (2) reduce to $E(\hat{\sigma}^2_{\beta_k}) = \sigma^2_{\beta_k}$ and $E(\hat{\sigma}_{\beta_{kk'}}) = \sigma_{\beta_{kk'}}$, respectively.
