# Supplementary material for: A new approach fits multivariate genomic prediction models efficiently
Source: Genet Sel Evol. 2022 Jun 17;54:45. doi: 10.1186/s12711-022-00730-w (PMC9204867; doi:10.1186/s12711-022-00730-w)
Supplement: Supplementary file 3 — Additional file 3. Scenario 1 with more environments. [file 12711_2022_730_MOESM3_ESM.pdf]

Scenario 1 with more environments

Alencar Xavier and David Habier

May 6, 2022

Figure 1 of the main manuscript showed that PEGS and THGS were not better than UV-THGS in scenario 1 when both heritability and genetic correlations were low. With increasing number of environments, and thereby phenotypes per individual, PEGS and THGS had a higher accuracy than UV-THGS, and their accuracy approaches that of REML (Figure 1).

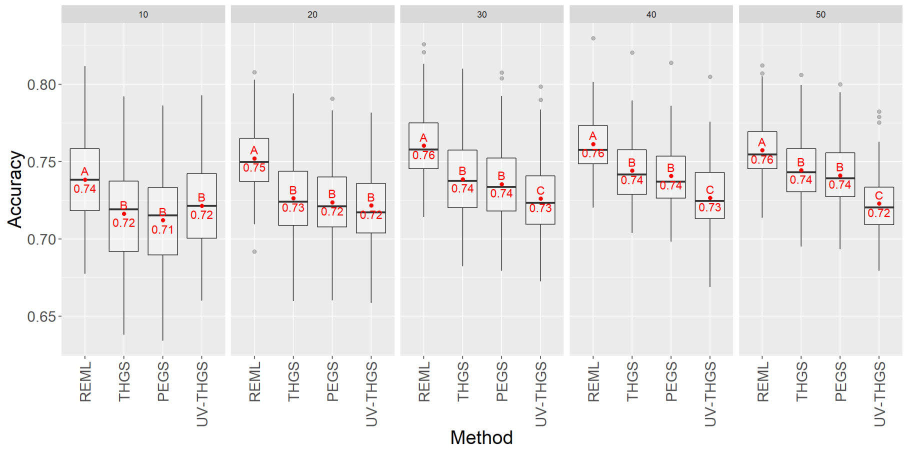

Figure 1: Accuracy of GEBVs in scenario 1 with an increasing number of environments (10, 20, 30, 40 and 50), heritability $h^2 = 0.2$ and low genetic correlations $(0.2 - 0.4)$.
