# Supplementary material for: A new approach fits multivariate genomic prediction models efficiently
Source: Genet Sel Evol. 2022 Jun 17;54:45. doi: 10.1186/s12711-022-00730-w (PMC9204867; doi:10.1186/s12711-022-00730-w)
Supplement: Supplementary file 4 — Additional file 4. Estimated covariances for different degrees of balanced data. [file 12711_2022_730_MOESM4_ESM.pdf]

Estimated covariances for different degrees of balanced data

Alencar Xavier and David Habier

May 6, 2022

REML results were not provided for scenario 2 because ASREML and AIREMLF90 were not suited for the estimation of covariance components when there was no overlap of individuals and environments. In Figure 1, the data from scenario 2 were adapted to demonstrate that an overlapping of individuals and environments is necessary for REML with ASREML and AIREMLF90 to adequately estimate covariance components. This pitfall was not a problem for PEGS and THGS.

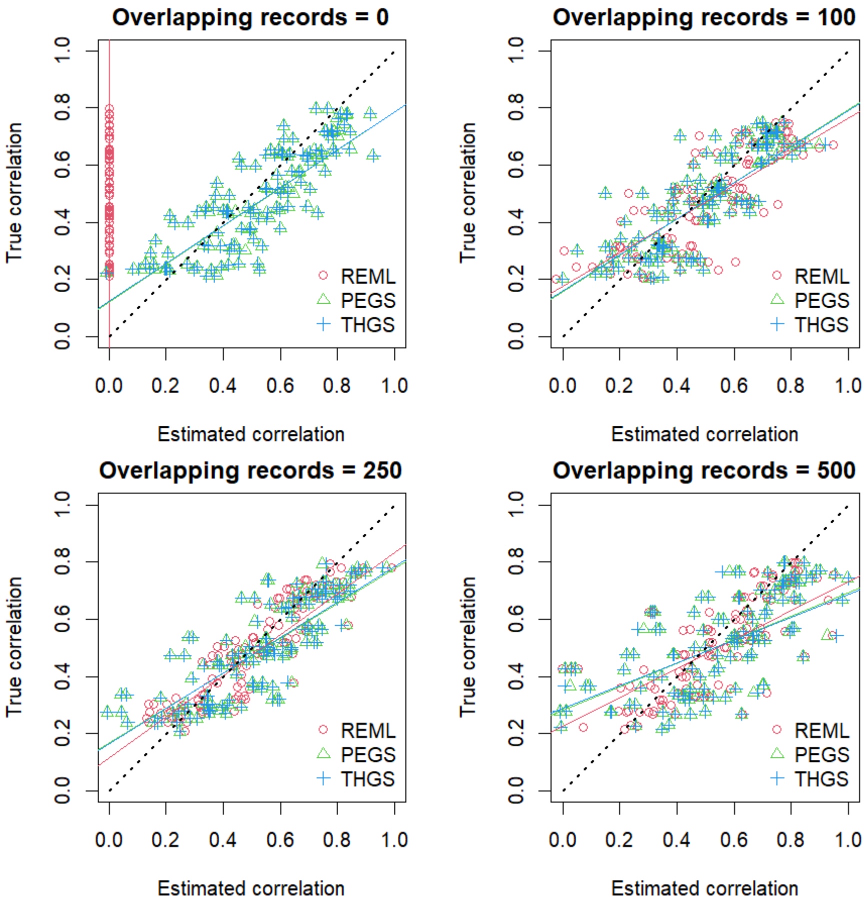

Figure 1: Scatter plot between true and estimated genetic correlations using the soybean dataset with varying number of overlapping individuals across environments.
