# Supplementary material for: A new approach fits multivariate genomic prediction models efficiently
Source: Genet Sel Evol. 2022 Jun 17;54:45. doi: 10.1186/s12711-022-00730-w (PMC9204867; doi:10.1186/s12711-022-00730-w)
Supplement: Supplementary file 5 — Additional file 5. Summary of scenario 2 and balanced case. [file 12711_2022_730_MOESM5_ESM.pdf]

Summary of scenario 2 and balanced case

Alencar Xavier and David Habier

May 6, 2022

Table 1: Accuracy of GEBVs, regression of TBV on GEBV (Slope) and bias of estimated heritabilities ($\hat{h}^2$) and genetic correlations (GC) using the soybean dataset for unbalanced (scenario 2) and balanced datasets (all response variables observed in all individuals) with varying number of observations per environment (Obs/Env). Based on 100 replicates of the simulation.

Scenario	Obs/Env	Method	Accuracy	Slope	Bias $\hat{h}^2$	Bias of GC
Unbalanced	514	PEGS	0.88 (0.02)	1.00 (0.03)	-0.01 (0.03)	0.00 (0.06)
Unbalanced	514	THGS	0.88 (0.02)	1.00 (0.03)	-0.01 (0.03)	-0.01 (0.06)
Unbalanced	514	UV-THGS	0.86 (0.03)	1.03 (0.05)	-0.01 (0.03)	-
Balanced	5,142	REML	0.97 (0.00)	1.00 (0.01)	0.00 (0.01)	0.00 (0.03)
Balanced	5,142	PEGS	0.97 (0.00)	1.00 (0.01)	-0.01 (0.02)	0.00 (0.05)
Balanced	5,142	THGS	0.97 (0.00)	1.00 (0.01)	0.00 (0.02)	0.00 (0.03)
Balanced	5,142	UV-THGS	0.96 (0.01)	1.00 (0.01)	0.00 (0.02)	-
