# Supplementary material for: A new approach fits multivariate genomic prediction models efficiently
Source: Genet Sel Evol. 2022 Jun 17;54:45. doi: 10.1186/s12711-022-00730-w (PMC9204867; doi:10.1186/s12711-022-00730-w)
Supplement: Supplementary file 6 — Additional file 6. R code demonstrating unbiasedness of PEGS. [file 12711_2022_730_MOESM6_ESM.pdf]

R code demonstrating unbiasedness of PEGS

Alencar Xavier and David Habier

May 6, 2022

The **input** variables are the marker scores, the additive-genetic variance of environment k, the additive-genetic variance of environment k', the additive-genetic correlation between the two environments, the residual variance of environment k, the residual variance of environment k', the number of individuals in environment k, and the number of individuals in environment k'. The marker scores are obtained from the R package SoyNAM, and matrices $\mathbf{Z}_k$ and $\mathbf{Z}_{k'}$ are populated with random samples of individuals from that dataset. The **output** is a vector that contains the heritabilities of the two environments, true additive-genetic variances and covariances, and the expected values and biases of their estimates.

```
# Function to estimate PEGS bias
#install.packages("SoyNAM")
require(Matrix)
bias = function(x,                # Genotypic information matrix
                sigma2_gk = 1,    # Additive genetic variance for environment 1
                sigma2_gkp = 2,   # Additive genetic variance for environment 2
                corr_gkkp = 0.7,  # Additive genetic correlation
                sigma2_ek = 5,    # Residual variance for environment 1
                sigma2_ekp = 7,  # Residual variance for environment 2
                nk=100,           # Number of individuals in environment 1
                nkp=110){         # Number of individuals in environment 2
  sigma_gkkp = corr_gkkp*sqrt(sigma2_gk*sigma2_gkp)
  n   = nk+nkp # Total number of individuals
  s = sample(nk+nkp,replace = F) # Sample random individuals
  Zk = x[s[1:nk],] # Marker scores of environment k
  Zkp = x[s[(nk+1):n],] # Marker scores of environment kp
  m = ncol(Zk) # Number of markers
  Zk = apply(Zk,2,function(x) x-mean(x)) # Centering of marker scores
  Zkp = apply(Zkp,2,function(x) x-mean(x)) # Centering of marker scores
  sigma2_betak  = sigma2_gk/m  # Marker variance for environment k
  sigma2_betakp = sigma2_gkp/m # Marker variance for environment kp
  sigma_betakkp = sigma_gkkp/m # Marker covariance between environments k and kp
  ZGZ = tcrossprod(bdiag(list(sqrt(sigma2_betak)*Zk,sqrt(sigma2_betakp)*Zkp)))
  ZGZ = as.matrix(ZGZ); ZGZ[1:nk,(nk+1):n] = sigma_betakkp*Zk%*%t(Zkp);
```

```r
  ZGZ[(nk+1):n,1:nk] = sigma_betakkp*Zkp%*%t(Zk)
  R = bdiag(list(Diagonal(nk,sigma2_ek),Diagonal(nkp,sigma2_ekp)))
  V = ZGZ + as.matrix(R); invV = solve(V)
  X = as.matrix(Matrix::bdiag(list(matrix(1,nk),matrix(1,nkp))))
  P = invV%*%(diag(n)-X%*%solve(t(X)%*%invV%*%X)%*%t(X)%*%invV)
  Mk  = diag(nk)-matrix(1/nk,nk,nk)
  Mkp = diag(nkp)-matrix(1/nkp,nkp,nkp)
  Vky  = cbind(sigma2_betak*t(Zk),sigma_betakkp*t(Zkp))
  Vkpy = cbind(sigma_betakkp*t(Zk),sigma2_betakp*t(Zkp))
  trZkMkZk = sum(diag(t(Zk)%*%Mk%*%Zk)) #tr(Zk*Mk*Zk)
  trZkpMkpZkp = sum(diag(t(Zkp)%*%Mkp%*%Zkp)) #tr(Zkp*Mkp*Zkp)
  ExpVal_tildeBetak_hatBeta_k  = sum(diag(t(Zk)%*%Mk%*%V[1:nk,1:n]%*%P%*%t(Vky)))
  ExpVal_tildeBetak_hatBeta_kp = sum(diag(t(Zk)%*%Mk%*%V[1:nk,1:n]%*%P%*%t(Vkpy)))
  ExpVal_tildeBetakp_hatBeta_k = sum(diag(t(Zkp)%*%Mkp%*%V[(nk+1):n,1:n]%*%P%*%t(Vky)))
  ExpVal_tildeBetakp_hatBeta_kp = sum(diag(t(Zkp)%*%Mkp%*%V[(nk+1):n,1:n]%*%P%*%t(Vkpy)))
  ExpVal_sigma2_gk_hat  = m*(ExpVal_tildeBetak_hatBeta_k)/trZkMkZk
  ExpVal_sigma2_gkp_hat  = m*(ExpVal_tildeBetakp_hatBeta_kp)/trZkpMkpZkp
  covSS = m*(ExpVal_tildeBetak_hatBeta_kp+ExpVal_tildeBetakp_hatBeta_k)
  ExpVal_sigma_gkkp_hat = covSS/(trZkMkZk+trZkpMkpZkp)
  bias_sigma2_gk_hat  = ExpVal_sigma2_gk_hat - sigma2_gk
  bias_sigma2_gkp_hat  = ExpVal_sigma2_gkp_hat - sigma2_gkp
  bias_sigma_gkkp_hat = ExpVal_sigma_gkkp_hat - sigma_gkkp
  h2_k = sigma2_gk/(sigma2_gk+sigma2_ek)
  h2_kp = sigma2_gkp/(sigma2_gkp+sigma2_ekp)
  ExpVal_Corr = ExpVal_sigma_gkkp_hat / sqrt(ExpVal_sigma2_gk_hat*ExpVal_sigma2_gkp_hat)
  bias_corr = ExpVal_Corr - corr_gkkp
  out = c(H2_k = h2_k, H2_kp = h2_kp,
          Sigma2_gk = sigma2_gk, ExpVal_sigma2_gk_hat = ExpVal_sigma2_gk_hat,
          Bias_sigma2_gk_hat = bias_sigma2_gk_hat,
          Sigma2_gkp = sigma2_gkp, ExpVal_sigma2_gkp_hat = ExpVal_sigma2_gkp_hat,
          Bias_sigma2_gkp_hat = bias_sigma2_gkp_hat,
          Corr_gkkp = corr_gkkp, ExpVal_Corr_gkkp = ExpVal_Corr,
          Bias_Corr_gkkp = bias_corr,
          Sigma_gkkp = sigma_gkkp, ExpVal_sigma_gkkp_hat = ExpVal_sigma_gkkp_hat,
          Bias_sigma_gkkp_hat= bias_sigma_gkkp_hat)
  return(round(out,4))}

# Get soybean data
x = SoyNAM::ENV()$gen

# Run example
bias(x)
```
